# Direct interactions with commensal streptococci modify intercellular communication behaviors of *Streptococcus mutans*

**DOI:** 10.1038/s41396-020-00789-7

**Published:** 2020-09-30

**Authors:** Justin R. Kaspar, Kyulim Lee, Brook Richard, Alejandro R. Walker, Robert A. Burne

**Affiliations:** 1 Department of Oral Biology, College of Dentistry, University of Florida, Gainesville, FL USA; 2 Division of Biosciences, College of Dentistry, Ohio State University, Columbus, OH USA

**Keywords:** Biofilms, Microbial ecology, Bacteriology, Bacterial genetics

## Abstract

The formation of dental caries is a complex process that ultimately leads to damage of the tooth enamel from acids produced by microbes in attached biofilms. The bacterial interactions occurring within these biofilms between cariogenic bacteria, such as the mutans streptococci, and health-associated commensal streptococci, are thought to be critical determinants of health and disease. To better understand these interactions, a *Streptococcus mutans* reporter strain that actively monitors cell–cell communication via peptide signaling was cocultured with different commensal streptococci. Signaling by *S. mutans*, normally highly active in monoculture, was completely inhibited by several species of commensals, but only when the bacteria were in direct contact with *S. mutans*. We identified a novel gene expression pattern that occurred in *S. mutans* when cultured directly with these commensals. Finally, mutant derivatives of commensals lacking previously shown antagonistic gene products displayed wild-type levels of signal inhibition in cocultures. Collectively, these results reveal a novel pathway(s) in multiple health-associated commensal streptococci that blocks peptide signaling and induces a common contact-dependent pattern of differential gene expression in *S. mutans*. Understanding the molecular basis for this inhibition will assist in the rational design of new risk assessments, diagnostics, and treatments for the most pervasive oral infectious diseases.

## Introduction

Bacteria within multispecies communities grow and persist in complex environments by competing over scare resources such as nutrients and physical space [1, 2]. One potential survival tactic is the synchronization of individual bacteria within a given species to coordinate their response to competitors through communication networks involving cell–cell signaling [3, 4]. This process is often referred to as quorum sensing, as coordinated changes in gene expression patterns (GEP) are observed once the signal has reached a critical concentration that often correlates with the density of the bacterial population [5]. In recent years, considerable progress has been made in understanding the molecular mechanisms that govern bacterial cell–cell communication pathways, but the biology and significance of these systems have mainly been studied with monospecies cultures in reasonably well-defined conditions [6]. While this reductionist approach has yielded a wealth of information, current exploration of complex microbial populations supports the need to examine cell–cell signaling behaviors under conditions that more closely mimic the natural environment where the signaling may be of ecologic significance.

The human oral cavity is ideally suited as a model for the dissection of interspecies interactions. In the human mouth, billions of microbes belonging to over 700 independent taxa function cooperatively and/or antagonistically to shape the composition of the microbiome [7, 8]. These interactions, coupled with frequent environmental perturbations, can also disrupt microbial homeostasis; which can lead to the ecological shifts observed during development of oral diseases [9]. For example, as is the case for dental caries (tooth decay), increases in the proportions of strongly acidogenic and acid tolerant bacteria, such as the mutans streptococci, are observed when bacterial fermentation of dietary carbohydrates repeatedly acidifies microbial biofilms, leading to demineralization of the tooth [10–12]. Concurrently, health-associated commensal streptococci, which are less constitutionally resistant to low pH, decrease in proportions. Loss of these species compounds the problem as many of these commensal streptococci provide protection from caries development by metabolizing arginine via the arginine deiminase system [13, 14], which elevates the pH through release of ammonia. Furthermore, many commensal streptococci can directly inhibit growth and/or expression of virulence traits by mutans streptococci through multiple strategies; with the generation of hydrogen peroxide being a significant deterrent to growth of *Streptococcus mutans* [15, 16] and other oral pathogens [17]. Recently, it was shown that a novel *Streptococcus* strain, designated *Streptococcus* sp. A12, has the ability to degrade the two primary cell–cell signal peptides of *S. mutans*, competence stimulating peptide (CSP) and *comX-*inducing peptide (XIP) [18, 19], suggesting that inference of cell–cell communication could be an underappreciated antagonistic mechanism that contributes to caries resistance.

Gram-positive bacteria utilize short, hydrophobic peptides, sometimes termed pheromones, as signaling molecules to control a spectrum of processes, including genetic competence, sporulation and production of toxins [20]. Genetic competence, a transient phenotypic state that renders cells able to internalize extracellular DNA, has proven to be a particularly informative model to study cell–cell signaling by streptococci, with two distinct cell signaling systems present controlling competence induction in these organisms. One extracellular signaling system consists of a signal-sensing kinase on the cell surface that transduces signal perception to a cytoplasmic response regulator via phosphorylation. In streptococci, this pathway (ComCDE) has been most extensively studied in *Streptococcus pneumoniae* [21–23]. A second, intercellular signaling mechanism is present in other phylogenetic groups of streptococci, wherein the peptide signal is imported into the cell and is bound by a cytosolic transcriptional regulator that controls the activities of different promoters to regulate gene expression [24, 25]. The best described example of this system is the ComRS pathway of the dental caries pathogen *S. mutans* [26]. Here, the propeptide ComS is exported by select transporters [27] and by cell lysis [28, 29]. The mature, active peptide, XIP, accumulates extracellularly [30] and can be reimported into the cell by the Opp oligopeptide permease [31]. XIP is bound by the cytosolic, Rgg-type regulator ComR [32], leading to conformational changes that allow the dimeric ComR-XIP complex to activate *comS* transcription [24], creating a positive feedback loop for the system. The ComR-XIP complex also activates the expression of the sole alternative sigma factor in *S. mutans*, *comX*, that controls competence development through activation of multiple genes; including those for the production of a pilus that uptakes exogenous DNA and for homologous recombination of the internalized single-stranded DNA [33–35]. Other ComRS-like pairs are encoded in multiple streptococci, where the cognate small hydrophobic peptide (SHP) encoded by the *comS*-like gene interacts with its cognate ComR-like transcriptional regulator, although there appears that certain SHPs may interact with multiple ComR-like proteins [36, 37]. The ComRS system of *S. mutans*, however, appears highly specific for *S. mutans* and genetic competence. Of note, cytoplasmic XIP and perhaps ComS may activate the pathway absent export and reimportation [29].

Previously, we provided definitive evidence that the XIP peptide of *S. mutans* can function as an intercellular signaling molecule to mediate ComRS cell–cell signaling and activation of genetic competence [28]. These experiments were performed in monospecies cocultures containing a genetically engineered *S. mutans* “sender” strain that overproduced XIP and an *S. mutans* “receiver” strain that lacked *comS*; signaling was measured in the *comS* mutant using a fluorescent reporter gene (*gfp*) fused to the *comX* promoter (P*comX*). The sender strain efficiently activated *comX* expression in the receiver strain, both planktonically and within biofilm populations. Here, we begin to describe how ComRS signaling is impacted by the presence of commensal streptococci, a closer approximation of the complexities of interactions that occur during the maturation of dental biofilms.

## Results

### Inhibition of cell signaling by commensal streptococci

**Fig. 1:**
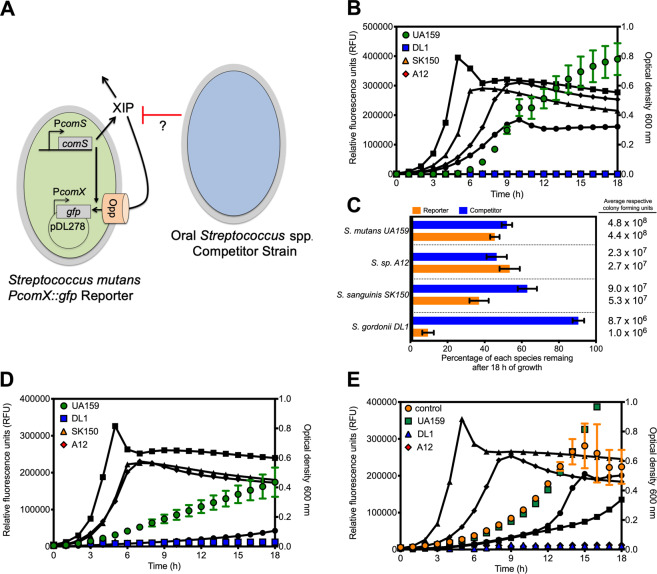
Loss of *S. mutans* peptide signaling in presence of competitor. **a** An oral *Streptococcus* spp. competitor strain (blue) was cocultured in chemically defined medium (CDM) with an *S. mutans* P*comX*::*gfp* reporter strain (green). As cell density of the reporter strain increases during growth, the XIP peptide that originates from the *comS* gene will be produced and accumulates extracellularly. XIP is then reimported into the cell through the Opp oligopeptide permease, binds to ComR and activates the *comX* promoter. Additionally, intracellular signaling occurs with ComS binding directly to ComR. The reporter strain harbors a plasmid, pDL278, carrying a copy of *gfp* that is driven by the *comX* promoter (P*comX*) to monitor ComRS signaling activation. **b** Cocultures of the *S. mutans* P*comX*::*gfp* reporter strain grown with either *S. mutans* UA159 (control, green circles), *S. gordonii* DL1 (blue squares), *S. sanguinis* SK150 (orange triangles), or *S*. sp. A12 (red diamonds). Colored, non-connected symbols represent relative fluorescent units (RFUs) plotted on the left *y*-axis, while black, connected lines with symbols represent growth of the cocultures over the course of the experiment measured by optical density at 600 nm plotted on the right *y-*axis. Data are averages from three biological replicates of the experiment. **c** Percentage of each species remaining within the coculture after 18 h of monitoring, determined by colony forming unit (CFU) plating. The P*comX*::*gfp* reporter strain is represented in the orange bars, while the competitor, listed on the left *y*-axis, is represented in blue. Average of collected CFUs is shown to the right. Data represent averages from three biological replicates of the experiment that was conducted in panel (**b**). **d** Cocultures of the *S. mutans* P*comX*::*gfp* reporter strain in which 5 µM sXIP was added prior to the start of the experiment. **e** Cocultures of the *S. mutans* P*comX*::*gfp* reporter strain that contains a plasmid that overexpresses the XIP peptide precursor, ComS. Control represents the P*comX*::*gfp* reporter that contained an empty vector only.

To study how *S. mutans* ComRS signaling could be impacted by the presence of a competing species, we empirically optimized a dual-species model system (Fig. 1a) in which a strain of *S. mutans* carrying the promoter regions of *comS* or *comX* (P*comS*, P*comX*) fused to a codon-optimized green fluorescence protein (*gfp*) reporter gene could be cocultured with wild-type strains of *Streptococcus gordonii* DL1, *Streptococcus sanguinis* SK150, or *S.* sp. A12. All experiments were performed in chemically defined medium (CDM) [38, 39] because activation of the ComRS circuit occurs spontaneously in CDM as cell density increases, with no need for addition of synthetic XIP or overexpression of the gene for the XIP precursor (*comS*) (Supplementary Fig. 1). CDM is also heavily buffered with phosphate, which is advantageous because ComRS signaling is optimal at neutral pH values [40, 41]. The buffer also prevents the generation of strongly acidic conditions by *S. mutans*, which is detrimental to the comparatively acid-sensitive commensal *Streptococcus* spp.

When the P*comX*::*gfp* reporter strain was cocultured with wild-type *S. mutans* UA159 (control), robust ComRS signaling was observed as cell density increased (Fig. 1b). However, when cocultured with a competitor *Streptococcus* spp., no signal from the *S. mutans* reporter could be detected above background levels; i.e., the nonspecific fluorescence generated by an *S. mutans* strain that did not contain a copy of the *gfp* gene. The lack of fluorescence in the cocultures with commensals was not due to growth inhibition of *S. mutans* as the reporter strain constituted 10 ± 3%, 37 ± 5%, or 54 ± 3% of the total colony forming units (CFUs) recovered after 18 h of coculturing with *S. gordonii* DL1, *S. sanguinis* SK150, or *S*. sp. A12, respectively (Fig. 1c). The quantity of *S. mutans* cells in the commensal cocultures compared favorably with the recovery of the reporter strain (54 ± 5%) in coculture with wild-type *S. mutans* UA159. Of note, the fact that equal proportions of reporter and wild-type *S. mutans* were recovered from cocultures demonstrated that the presence of the GFP gene fusion did not compromise the fitness of the reporter strain, further verified by growth rate comparisons between wild-type and reporter strains (Supplementary Fig. 1).

Two strategies were implemented to try to recover active ComRS signaling by the reporter strain during cocultivation with commensal streptococci. First, synthetic XIP was added to the cocultures to a final concentration of 5 µM just prior to the beginning of the fluorescence monitoring phase of the experiments, and cocultures were observed as above. No detectable fluorescence signal was recorded above background in the cocultures, with or without exogenously added XIP (Fig. 1d). Second, a plasmid encoding a copy of the XIP precursor *comS* under the control of a highly expressed constitutive promoter (P23) [42] was introduced into the *S. mutans* reporter strain; we previously reported that overexpression of *comS* could strongly activate P*comX* [28]. However, no increase in GFP expression was observed in cocultures of the *comS* overexpressing strain with the commensals, whereas signaling was greatly enhanced when cocultured with strain UA159 as a control (Fig. 1e).

**Fig. 2:**
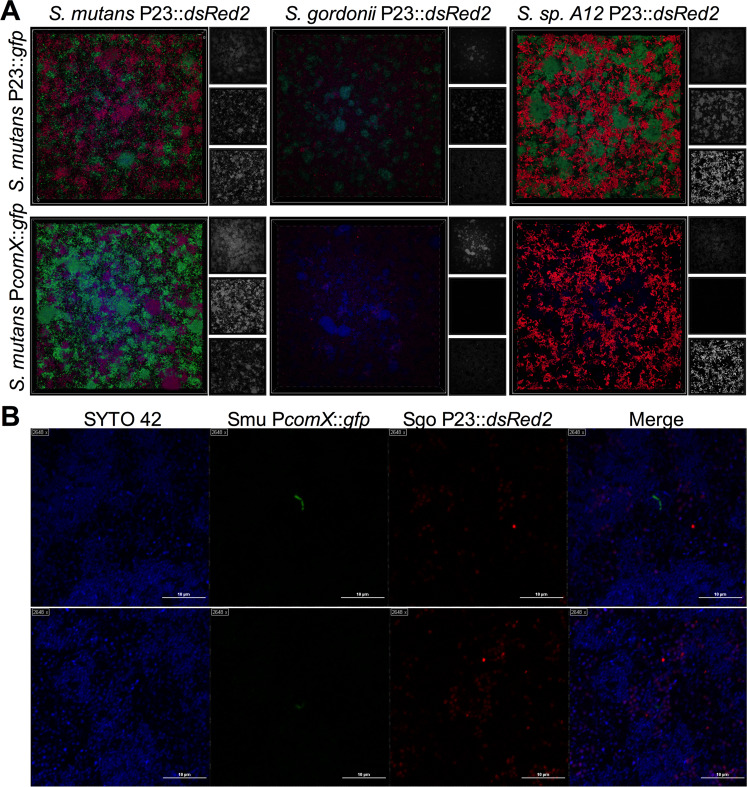
*S. mutans* peptide signaling in coculture biofilms. **a** 3D volume projections of imaged biofilms in the XY-orientation (from the top looking down). Each biofilm contains either *S. mutans* UA159 with a constitutive *gfp* reporter plasmid (top row), or the P*comX*::*gfp* reporter plasmid (bottom row) that was cocultured with either *S. mutans* (control; left), *S. gordonii* DL1 (middle), or *S.* sp. A12 (right) who all constitutively produce DsRed2. To the right of each expanded color image is the black and white image capture of each individual channel: blue (top), green (middle), and red (bottom). **b** Zoomed image frames of *PcomX*-active cells within cocultured biofilms with *S. gordonii* DL1. The images captured are a single *z* plane near or at the biofilm substratum. Two different areas of the biofilm (top and bottom rows) were imaged. Each panel represents one color channel of blue (SYTO 42 stained; total cells), green (P*comX*::*gfp* positive cells), or red (*S. gordonii* P23::DsRed2) followed by the merged image on the far right. The top panel of (**b**) is the same area of biofilm shown in Movie S1.

To ensure these observations were not limited to only planktonic growth conditions, we examined *S. mutans* ComRS signaling in cocultured biofilm populations. While almost all cells harboring the P*comX*::*gfp* reporter were GFP-positive in the control biofilms (coculture of the reporter with wild-type *S. mutans*), confocal imaging of biofilms containing competitor streptococci uniformly showed that almost no *S. mutans* cells were expressing detectable GFP (Fig. 2a). However, in some frames (0.22 × 0.22 mm frames, ~30,000 *S. mutans* cells per frame), a small number of cells (1–3 cells per frame) were GFP-positive. When 3D renderings of these areas within the biofilm were constructed, GFP-positive cells were found close to the substratum (Fig. 2b and Movie S1, same area of biofilm as top panel of Fig. 2b). Also, P*comX-*active cells were not necessarily confined to distinct *S. mutans* microcolonies, and in some cases could be seen adjacent to the competitor streptococci, which carried a constitutively expressed red fluorescent protein (DsRed2) for their identification. To quantify the different types of cells in the biofilm populations, we physically dispersed the biofilms by sonication and analyzed the populations by flow cytometry (Supplementary Fig. 2). About 1 in 10,000 *S. mutans* cells counted displayed activation of P*comX* within the biofilms, which was similar to the proportions of GFP-expressing cells in planktonic growth conditions (Supplemental Table 1).

### Commensal signaling inhibition is dependent on cell contact

**Fig. 3:**
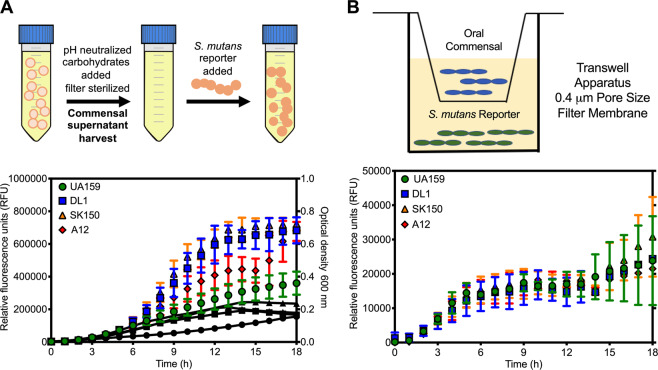
Cell contact dependence in signaling inhibition. **a** Growth and fluorescence of *S. mutans* P*comX*::*gfp* reporter strain in spent supernatant fluids of either *S. mutans* UA159 (control, green circles), *S. gordonii* DL1 (blue squares), *S. sanguinis* SK150 (orange triangles), or *S.* sp. A12 (red diamonds). Depiction on top shows methods used to treat supernatant fluids following harvesting and prior to reporter strain inoculation. Overnight cultures of selected strains where centrifuged, spent supernates removed, filter sterilized, the pH was adjusted to 7.0 and 20 mM additional glucose was added. The P*comX*::*gfp* reporter strain was then inoculated and monitored for 18 h in a Synergy 2 multimode plate reader. **b** Growth of cocultures in a transwell apparatus. The P*comX*::*gfp* reporter strain was first inoculated in 0.1 mL of CDM medium in a 96-well microtiter plate. The transwell plate was then overlaid on top of the 96-well plate, and 0.1 mL of CDM inoculated with either *S. mutans* UA159 (control, green circles), *S. gordonii* DL1 (blue squares), *S. sanguinis* SK150 (orange triangles), or *S.* sp. A12 (red diamonds) was added to the top chamber, as shown. Cultures of the reporter strain and competitor were separated by a 0.4 µM pore size polycarbonate filter membrane. Fluorescence (RFUs) of the reporter strain was monitored for 18 h.

Changes in phenotypes that are observed when two different species of bacteria are cocultured can usually be induced by secreted molecules from one of the bacterial strains [1]. We suspected that molecule(s) secreted by the competitor strains are required for shutting down cell–cell signaling in *S. mutans*. To explore this hypothesis, we cultured the competitors individually overnight and collected the supernatant fluids after centrifugation. The supernates were then filter sterilized, pH adjusted from ~6.3 to 7.0 with NaOH, and carbohydrate was added back to achieve a final concentration of added glucose to 20 mM. We then inoculated our reporter strain into the commensal supernates and monitored fluorescence activity (Fig. 3a). Surprisingly, ComRS signaling was readily observed in all supernates. In fact, reporter activity tended to be higher in the supernates of competitors compared to controls.

In another experiment to confirm these results, we grew competitor and our reporter strains together in a transwell apparatus, so that both bacterial strains shared the same growth medium, but were physically separated by 0.4 µm pore size polycarbonate membrane that would allow passage of small molecule(s) between the chambers (Fig. 3b). Even in the transwell system, cell signaling was robust in cocultures containing competitor species. This result is consistent with data showing that the proximity of live commensal cells with *S. mutans* prior to signal activation is required for the signaling inhibition.

### Impairment of *S. mutans* cell signaling by oral commensals is conserved across species

**Fig. 4:**
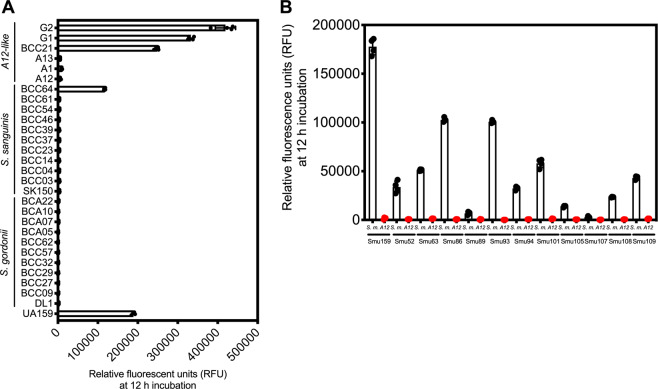
Conservation of ComRS signaling antagonism across oral isolates. **a** Relative fluorescent units (RFUs) of the *S. mutans* P*comX*::*gfp* reporter strain cocultured with clinical oral isolates of either *S. gordonii*, *S. sanguinis* or *S*. sp. A12-like strains. Relative fluorescent units were recorded after coculture inoculation at 1:1 ratio and 12 h of incubation at 37 °C. Results from four biological replicates of the experiment are shown. **b** RFUs after 12 h of incubation of the P*comX*::*gfp* reporter harbored in various *S. mutans* clinical isolates. The P*comX*::*gfp* reporter strain was cocultured with either *S. mutans* UA159 (control; black dots and bars) or an oral competitor streptococci (*S.* sp. A12, red dots and bars).

We next screened a collection of low-passage oral streptococci that had been previously genome sequenced [43] to determine whether the ability to inhibit *S. mutans* ComRS signaling was conserved across commensal species and to assess whether the presence or absence of certain genes might contribute to inhibition of peptide signaling. Ten different low-passage clinical isolates of *S. gordonii*, ten isolates of *S. sanguinis*, and five isolates of *S*. sp. A12-related organisms [19] were cocultured with our *S. mutans* ComRS signaling reporter. The *S*. sp. A12-related organisms included strains classified as A12-like (A13 and BCC21), as *Streptococcus australis* (G1 and G2), or as *Streptococcus parasanguinis* (A1). Interestingly, significant production of GFP by *S. mutans* was evident when cultured with one isolate of *S. sanguinis* (BCC64) and with three isolates that were classified as A12-related (BCC21, G1 and G2) (Fig. 4a). However, these results were most likely due to the inability of these isolates to grow well within the CDM medium during the course of the experiment (Supplementary Fig. 3). In fact, after 18 h of monitoring, these isolates comprised <0.01% of the total CFUs recovered. Conversely, all commensal strains that grew well in CDM (achieved an OD_600_ > 0.1 after 12 h as monitored using a Bioscreen system, see Supplementary Fig. 3) inhibited P*comX* activation. Thus, if a commensal strain could grow in CDM, even somewhat poorly, it could completely inhibit ComRS signaling.

We also tested several genomically and phenotypically diverse isolates of *S. mutans* [44, 45], both in coculture with our P*comX*::*gfp* reporter in the UA159 background (Supplementary Fig. 4) and against competitor *Streptococcus* spp., after transformation of the *S. mutans* strains with the P*comX* reporter plasmid (Fig. 4b). Various levels of spontaneous activation of the P*comX*::*gfp* reporter were observed among the different *S. mutans* strains in monocultures in CDM, consistent with recent reports showing strain-dependent differences in *S. mutans* peptide signaling [46]. One isolate, Smu107 (R221), had undetectable levels of GFP in monoculture in CDM alone. All others showed activity above baseline. However, when cocultured with *S*. sp. A12, ComRS signaling was inhibited to an extent similar to that observed with strain wild-type UA159. Therefore, the ability to obstruct ComRS signaling is conserved among isolates of *S. gordonii*, *S. sanguinis*, and A12-related streptococci, and inhibition by commensals is similarly conserved in genomically diverse isolates of *S. mutans*.

### Relatively small proportions of live commensal streptococci can inhibit signaling

**Fig. 5:**
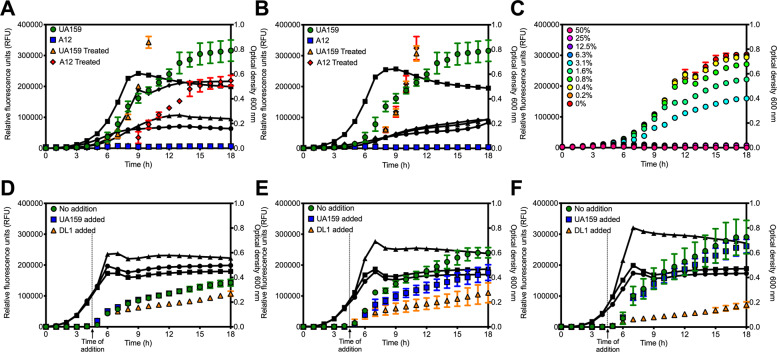
Importance of oral competitor cell density in signaling inhibition. Cocultures of the *S. mutans* P*comX*::*gfp* reporter strain with untreated or treated cells by either **a** 0.5 h heat inactivation at 80 °C or **b** 1 h suspension in 4% paraformaldehyde. Data represent averages from three biological replicates. **c** Dilution of an oral competitor streptococci (*S.* sp. A12) in coculture with the *S. mutans* P*comX*::*gfp* reporter strain. Legend (top left) refers to the amount of *S.* sp. A12 within the coculture at the time of initial inoculation. Bottom: addition of either control (UA159; blue squares) or an oral competitor streptococci (*S. gordonii* DL1; orange triangles) at 4.5 h to a growing culture of the *S. mutans* P*comX*::*gfp* strain when competence activation was **d** fully detected, **e** beginning to be detected, or **f** not yet detected. See Supplementary Fig. 7 for comparisons at 4.5 and 12 h, specifically.

To verify that the ability of the competitor species to grow (viability) was required for inhibition of peptide signaling, we used two different treatments of the competitor species *S*. sp. A12 after it was grown to mid-exponential phase: 80 °C for 0.5 h in a heating block (Fig. 5a) or treatment with 4% paraformaldehyde for 1 h at ambient temperature (Fig. 5b). After treatment, the inactivated commensal cells were washed and resuspended in fresh CDM and then mixed with the *S. mutans* reporter strain to begin the experiment. With heat-treated cells, some ComRS signal activity was evident, but not near the levels seen with *S. mutans-*only controls. However, when the paraformaldehyde-treated cells were used, the competitor did not inhibit signaling and fluorescence, with levels being similar to the *S. mutans-*only control. Importantly, we determined that there was a greater number of live cells, by plating and counting CFUs, for the competitor after heat treatment, compared to paraformaldehyde fixing (Supplementary Fig. 5), which likely explains the difference in effects on P*comX* activation. These results support that metabolically active and growing competitors are required for *S. mutans* ComRS signaling obstruction.

Based on the intermediate inhibitory effects seen with reduced proportions of a live competitor on our reporter strain, i.e. with heat-treated cells, we tested whether some minimal proportion of live competitor was required to exert effects on ComRS signaling. We utilized *S*. sp. A12 and varied the percentage of *S. mutans* and *S*. sp. A12 in the cocultures, after determining that the proportions of cells recovered after 18 h were similar to the proportions in the initial inocula (Supplementary Fig. 6). Complete inhibition of *S. mutans* ComRS signaling occurred when *S*. sp. A12 constituted ≥6.3% of the initial inoculum (Fig. 5c). At 3.1 or 1.6% of *S*. sp. A12, reporter activity was detectable, but at lower levels than when no *S*. sp. A12 was present. No difference in *S. mutans* reporter activity was observed when <1% of the inoculum was *S*. sp. A12.

Finally, we determined if timing of the introduction of the competitor commensal to the coculture affected peptide signaling behavior. In this experiment, we inoculated the reporter strain at three different dilutions (1:50, 1:66, and 1:100) to allow ComRS signaling to initiate at different time points during the incubation period, since activation requires that *S. mutans* attain a threshold cell density. Simultaneously, we began growing a culture of the competitor (for this experiment *S. gordonii* DL1) and control (*S. mutans* UA159) separate from our reporter strain. Fluorescence of the P*comX*::*gfp* reporter was actively monitored, and at a time point (4.5 h) when fluorescence was fully detected (Fig. 5d), beginning to be detected (Fig. 5e) or had not yet been detected (Fig. 5f) depending on the dilution used, the competitor was added and fluorescence activity and optical density were monitored (see Supplementary Fig. 7A). Interestingly, in the case where cell–cell signaling had already been activated (Fig. 5d), or was beginning to be detected (initial stages of ComRS activation) (Fig. 5e), addition of competitor did little to dampen reporter activity. However, when competitor was added at the time point when there was no evidence yet of activation (Fig. 5f), the presence of competitor significantly impaired *com* gene activation, as seen at the 12 h time point (Supplementary Fig. 7B).

### Transcriptome profiling of dual-species interactions

**Fig. 6:**
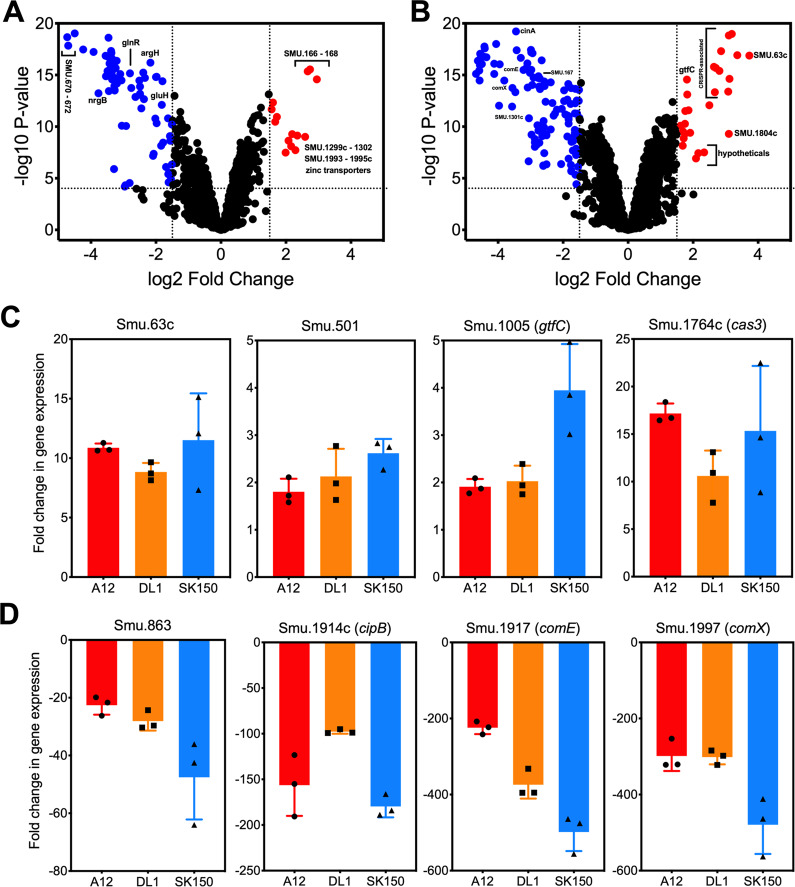
*Transcriptome Profiling S.mutans* culture in supernates or in direct contact. Volcano plots from transcriptome analysis of **a** *S. mutans* UA159 cultured in *S*. sp. A12 spent supernatant fluid compared to culturing in *S. mutans* UA159 spent supernatant fluid and **b** *S. mutans* UA159 directly cocultured with *S*. sp. A12 compared to culturing in *S*. sp. A12 spent supernatant fluid only. Data represent three independent replicates of each condition. Log2 fold changes and false discovery rates (FDR) converted to −log10 *p* values were calculated from the Degust website using edgeR analysis. Genes of interest that were ≥(−)1.5 log2 fold change and ≥4 −log10 *p* values were highlighted either in blue (downregulated, upper left quadrant) or red (upregulated, upper right quadrant) and are listed in Supplementary Tables 2 and 3, respectively. qRT-PCR confirmation of selected **c** upregulated or **d** downregulated genes from transcriptome analysis. Cocultures of *S. mutans* UA159 and selected competitors were grown in CDM medium to OD_600_ = 0.5 before harvesting for RNA extraction. Data represent fold change in gene expression compared to *S. mutans* UA159 monocultures. Three independent cocultures were analyzed and plotted.

To determine if the proximity-dependent effects on *S. mutans* of encountering oral competitor streptococci was confined to ComRS signaling and genetic competence activation, we performed transcriptome profiling by RNA-Seq of *S. mutans* grown in CDM under three different conditions: (1) growth in its own (*S. mutans*) spent supernatant fluid, (2) growth in spent supernates of a competitor (*S*. sp. A12; treated similarly to Fig. 3a), or (3) cocultured in fresh CDM medium directly with competitor (*S*. sp. A12). When comparing the growth of *S. mutans* in its own spent supernates against competitor spent supernates, we found 88 *S. mutans* genes differentially expressed (Log_2_ fold change ≥ (−)1.5, −log10 *p* value ≥ 4), which included upregulation of the zinc transport system and several amino acid ABC transporters, along with downregulation of the TnSmu1 genomic island (Fig. 6a and Supplementary Table 2). A more substantial effect was seen when we analyzed the transcriptomes of *S. mutans* grown in direct cocultivation with *S*. sp. A12 compare to *S*. sp. A12 supernates alone (Fig. 6b and Supplementary Table 3). In this case, 140 genes were differentially expressed in *S. mutans*, including upregulation of one of the CRISPR gene clusters and, as would be expected, downregulation of the entire genetic competence regulon in cells grown directly with *S*. sp. *A12*. Principal component analysis of transcriptome data from these three conditions displayed a wide separation of the tested groups, demonstrating that there is a unique transcriptomic response by *S. mutans* when it is grown directly with a competitor, as opposed to cultivation in spent supernates of the  competitor (Supplementary Fig. 8).

To determine if these transcriptomic responses were specific to competition with *S*. sp. A12 or represented a generalized response to  commensal streptococci, as well as to confirm our RNA-Seq data set, we performed qRT-PCR on harvested RNA from cocultures of *S. mutans* grown with either *S*. sp. A12, *S. gordonii* DL1, or *S. sanguinis* SK150, or from *S. mutans* grown in monoculture. Two unique core genes of *S. mutans* [44] that were not differentially expressed in our RNA-Seq experiment, SMU.996 and Smu.1616c, were used to normalize the amount of *S. mutans* present at the time of harvest between all cocultured samples (Supplementary Table 4). In total, eight genes were probed that represented both upregulated (Fig. 6c) and downregulated (Fig. 6d) genes found during our RNA-Seq experiment. Remarkably, *S. mutans* displayed the same genetic response in all three cocultures with different competitor species, including upregulation of *cas3* and the gene for a secreted glucosyltransferase (*gtfC*) required for sucrose-dependent biofilm formation [47, 48]. Aside from a significant decrease in *comX* expression, bacteriocin gene expression was also impacted in all three cocultures, including a decrease in *comE* (bacteriocin-related response regulator) and *cipB* (ComE-regulated bacteriocin). We propose that these selected probed genes in part represent potential larger transcriptomic changes of a conserved GEP by *S. mutans* to the presence of competitors that has not been previously reported.

### Inhibition of cell signaling is a novel antagonism mechanism by oral streptococci

**Fig. 7:**
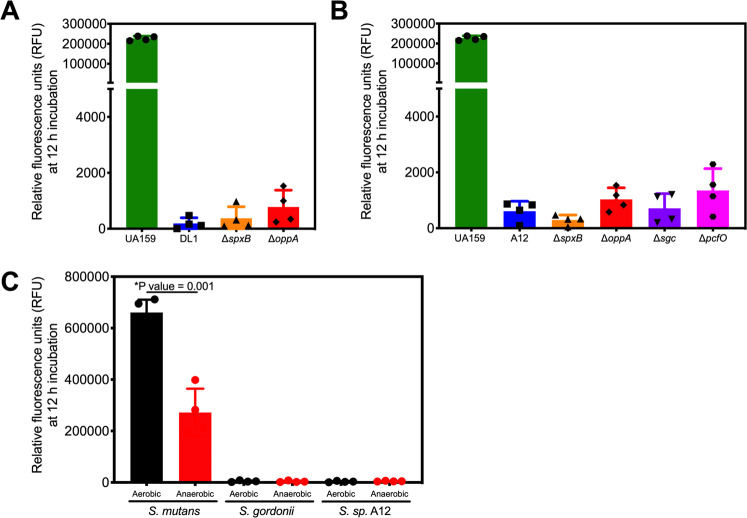
*Selected* competitor mutants still inhibit *S. mutans* peptide signaling. Cocultures of the *S. mutans* P*comX*::*gfp* reporter strain with mutants of selected oral commensal streptococci. Comparison of relative fluorescent units (RFUs) after 12 h of incubation in strains of **a** *S. gordonii* DL1, **b** *S.* sp. A12, and **c** in either aerobic (black bars) or anaerobic (red bars) conditions. Data are averages from four biological replicates of the experiment. The changes between parental and selected commensal mutants are all not significant. Statistical analysis shown in (**c**) was completed using Student’s *t* test.

Oral commensal streptococci, such as *S. gordonii* and *S. sanguinis*, antagonize *S. mutans* through different mechanisms, including hydrogen peroxide production [15, 16] and secretion of proteases that degrade signaling molecules [18, 19, 49]. To determine if these known antagonistic pathways were responsible for contact-dependent inhibition of *com* gene activation, we cocultured our reporter strain with *S. gordonii* (Fig. 7a) or with *S*. sp. A12 (Fig. 7b) that carried deletions in known genes involved in antagonism. Specifically, for hydrogen peroxide production, we tested competitors that carried a deletion of *spxB*, encoding pyruvate oxidase [16, 18]. No recovery of *S. mutans* ComRS signaling was observed when cocultured with the *spxB* mutants, suggesting hydrogen peroxide production was not required for obstruction of signaling. To further rule out the effects of oxygen metabolism as a potential mechanism for inhibition, we grew the cocultures anaerobically, yet saw no changes in ComRS signaling inhibition (Fig. 7c). In fact, reporter actively was significantly lower in anaerobic conditions, compared to the controls. Two different peptidases of *S*. sp. A12 degrade *S. mutans* signaling molecules. Sgc (an apparent orthologue of *S. gordonii* Challisin (49)) has CSP degrading activity [18], whereas PcfO of A12, encoded by an apparent orthologue of *pepO* in multiple *Streptococcus* spp., can degrade *S. mutans* XIP [19]. However, mutation of either gene had no significant effect on ComRS signaling interference in our P*comX* reporter assay. We additionally analyzed *S. mutans* gene expression via qRT-PCR with six differently expressed genes, as seen in Fig. 6, grown in coculture with *S*. sp. A12 and its mutant derivatives (Supplementary Fig. 9). We saw no significant changes in the upregulated genes among the cocultures with the *S*. sp. A12 mutants, but did see significant differences in *comX* gene expression (~1.9-fold increase) with A12∆*pcfO* and a ~16-fold increase in *comE* expression in the *S. mutans* coculture with A12∆*sgc*, suggesting both proteases do contribute to the dampening of *S. mutans* peptide signaling, albeit not with any apparent measurable effect on P*comX* induction based on the reporter assays. Finally, we also proposed that the competitors could be internalizing *S. mutans* XIP as a potential nutritional source in the peptide-free CDM medium. However, we saw no change when the *opp* homolog of *S. gordonii* (old NCBI locus tag SGO_1712) or of *S*. sp*. A12* (ATM98_08725) was deleted. Collectively, then, we conclude that there are novel and mechanistically uncharacterized strategies used by genetically diverse commensal streptococci to impair *S. mutans* cell–cell signaling that also lead to a unique transcriptome response that is distinct from monocultures or when *S. mutans* is exposed to supernates of commensal streptococci.

## Discussion

Recently our group reported that part of the arsenal deployed by *S*. sp. A12, a health-associated commensal with probiotic properties, to compete with the caries pathogen *S. mutans* includes its ability to degrade peptide cell–cell signaling molecules [18, 19]. In particular, incubation of synthetic CSP or XIP with only culture supernates alone of *S*. sp. A12 abolishes the peptides’ activity via Sgc or PcfO, respectively [18, 19]. We set out in this study to explore how ComRS signaling in *S. mutans* is altered during direct growth with these competitor species, conditions that should more closely mimic oral biofilms. We were immediately surprised to find that a peptide signaling system, usually displaying unimodal activation in our selected experimental conditions with cells grown in monoculture [31], was shut-off in all but a tiny fraction (1 in 10,000 cells) of the population by cocultivation with streptococcal isolates commonly found in supragingival plaque; inhibition was effective by both laboratory strains and by low-passage clinical isolates. Perhaps even more intriguing was the observation that the signaling inhibition occurred only when the strains were cocultured in ways that would permit direct cell-to-cell contact, and that the inhibition did not occur when *S. mutans* was growing in spent supernates from the commensals or when commensal and pathogen were separated in a transwell apparatus but shared the same growth medium. Mutants of A12 defective in the production of proteases that may degrade signal peptides of *S. mutans* retained wild-type levels of inhibition in cocultures. Collectively, these results support the existence of a novel mechanism(s) that requires direct contact between the species, both in planktonic growth conditions and within biofilms. While the apparent contact-dependent mechanism(s) appears to be dominant in terms of blocking ComRS-dependent gene activation, our findings still support a role for Sgc and PcfO in the inhibition of peptide signaling, as coculturing of *S. mutans* with *sgc* or *pcfO* mutants of *S*. sp. A12 resulted in significant alterations in expression of *S. mutans* genes controlled by signal peptides (Supplementary Fig. 9). However, these strains most likely have minimal impact within our reporter coculture assays reported here as they displayed wild-type levels of ComRS signaling inhibition and effects seen are almost entirely driven by the novel contact-dependent mechanism.

**Fig. 8:**
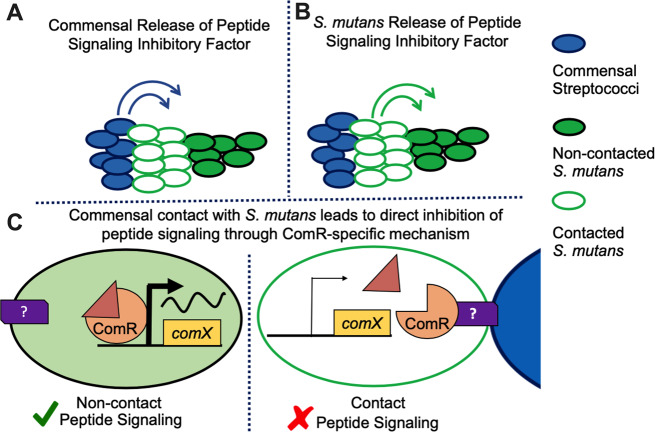
Model for *S. mutans* peptide signaling inhibition. Three different models of direct contact with commensal streptococci (blue cells) leading to *S. mutans* (direct contact white cells with green outline, noncontact green cells) peptide signaling inhibition and conserved gene expression patterns. **a** A commensal-derived inhibitory product from the initiation of contact with *S. mutans* is secreted to modify *S. mutans* behavior and peptide sensing across the entire *S. mutans* population. **b** A subset of *S. mutans* that is in direct contact with commensals produces an inhibitory signal that modifies behavior for the rest of the population, including those noncontacted *S. mutans* cells. **c** Transient contact between an oral commensal (blue) and *S. mutans* cell (green/green outline), prior to ComRS signaling activation, leads to posttranscriptional modifications in ComRS signaling. In the proposed mechanism shown, contact between *S. mutans* and commensal leads to sequestering of ComR with an unknown interaction partner, preventing ComR-XIP complex binding to the P*comX* promoter and genetic competence activation.

Based on our results and the current understanding of peptide signaling and control of genetic competence, we have developed three working models for how cell signaling inhibition is affected via direct contact of commensals with *S. mutans* (Fig. 8), only one of which is supported by the current data. The first set of observations that is central to the model is that commensals must be alive and in contact with *S. mutans*, whether in a planktonic or biofilm state, such that the potential for direct cell–cell contact between pathogen and commensal is possible, even if only transiently. It is also relevant that *S. mutans* biofilm communities are clustered in microcolonies [50–52], with recent intact imaging of multispecies biofilms on the diseased-tooth surface displaying a “dome-like” architecture, consisting of a densely packed *S. mutans* core with commensal streptococci overlaid on top [53], similar to the microcolonies seen in our coculture biofilm data here. The second observation that is central to our models is that the addition of exogenously supplied sXIP cannot overcome the inhibition exerted by commensals cultured in direct contact. Relevant to both observations, commensals cannot effectively block *comX* activation if the population has already initiated activation of the ComRS circuit. With those constraints, we exclude a handful of apparently simple explanations and arrive at a hypothesis that will be tested in future studies.

Based on existing literature, the simplest explanation is that the commensals all produce a factor(s) that blocks the activation of the ComRS circuit and induces a specific *S. mutans* GEP shown by our RNA-Seq results (Fig. 8a). Presumably, this would be a small molecule that can enter *S. mutans* and either complex with ComRS to shut down the ability of the XIP-ComR complex to activate its target gene(s) or lead to other transcriptomic responses resulting in similar effects. However, both the spent supernates and the transwell experiments argue that either this substance is not produced by, or is not released from the commensals, unless contact with *S. mutans* occurs. We do not favor this hypothesis because if we coculture *S. mutans* with the commensals, recover those supernates and then perform our reporter experiment in those supernates, there is no evidence of inhibition of P*comX* activity (Supplementary Fig. 10). Consistent with, but not definitively supporting this logic is the observation that gene expression in the commensal (*S*. sp. A12) is not substantially altered by cocultivation with *S. mutans* (K. Lee, manuscript in preparation); unlike *S. mutans*, which shows dramatic changes in gene expression after direct exposure to the commensals. Thus, it does not appear that growth in the presence of *S. mutans* activates the expression of a specific gene product in commensals that directly causes inhibition or synthesis of an inhibitory factor. While the RNA-Seq of the commensal does not rule out potential posttranscriptional activities, the commensal gene expression data coupled with the fact that supernates from cocultures of commensals with *S. mutans* do not inhibit *comX* activation seems to exclude that a diffusible factor produced and/or released by the commensals leads to a blockage of the ComRS circuit and the observed *S. mutans* GEP.

The second model we considered was one in which contact with the commensal triggered the expression and/or release of a signal by *S. mutans* that propagates through the population to exert a negative effect on ComRS-dependent activation and inducible GEP (Fig. 8b). While this model could help explain how such a small proportion (<2%) of commensals in cocultures could cause complete inhibition of *comX* activation, it is not consistent with the coculture supernate results mentioned above. We acknowledge that the hypothetical *S. mutans* signal molecule could be particularly labile or could work in concert with other cell-associated factors that would not be present in supernates, so we cannot completely exclude that *S. mutans* produces a signal that propagates through the population to shut off the ComRS circuit after contact with commensals, and retain the idea that such a factor could be necessary, but not sufficient.

The final working model is one we believe is most consistent with the data collected thus far, but it is also one that is not as common in terms of established interbacterial interactions. In particular, we posit that direct contact between the commensals and *S. mutans*, even in a relatively transient or brief fashion and as long as it is prior to activation of the ComRS circuit, induces changes in *S. mutans* that shut down the ComRS pathway (Fig. 8c). Importantly, there was no detectable alteration in the expression levels of *comR* in *S. mutans* cocultured with *S*. sp. A12 or other commensals, so we posit that the block occurs at the posttranscriptional, and probably at the posttranslational level. Moreover, since *comX* activation is blocked by cocultivation, the posttranslational interference likely involves either degradation of ComR, ComS, and/or XIP, or production of a molecule(s) that can complex with, for example, ComR and prevent its interaction with ComS or XIP, or inactivate the ComR-XIP complex. One such factor could be XrpA, which is encoded within the *comX* gene [54]. XrpA appears to negatively affect the ability of ComR to bind its targets [55]. However, a mutant that can produce ComX, but not XrpA, did not display any differences from the wild-type genetic background in the coculture reporter assay used here (data not shown). Thus, a mechanism in which inhibition occurs prior to *comS* activation, perhaps through direct binding of a factor to ComR that renders the protein non-functional seems most compatible with existing data.

We have shown through our transcriptomic analyses with *S*. sp. A12, and later confirmed in a limited set of probed genes via qRT-PCR with other commensal species, that *S. mutans* may have a specific GEP in response to cocultivation with a competitor. Further transcriptomic studies with more competitors are needed to support and verify these findings. We noticed several intriguing gene candidates for further study that are highly upregulated that could serve as a mechanism to downregulate competence activation, including the *cas3* CRISPR system (entire SMU.1753c–SMU.1764c region) and SMU.63c, recently shown to form amyloids that can alter biofilm architecture [56]. The identification of upregulated SMU.63c is intriguing not only from a functional aspect in its role during *S. mutans* microcolony formation, but this gene is adjacent to *comRS* (SMU.61) and transcribed in the opposite orientation. Use of gene-specific primers during cDNA generation rules out effects of readthrough from *comRS* transcription that would impact our interpretation of these results. The role these operons play, as well as others, in the fitness of *S. mutans* against competitors, as well as in potential inhibition of peptide sensing and alterations of the *S. mutans* transcriptome will be explored in future studies.

The fragility of ComRS activation is well documented as it is dampened by the presence of nonspecific peptide media constituents [31], proteases [19, 57], certain carbohydrates [57, 58], shifts in pH [40, 41], changes in (p)ppGpp levels [59], and now commensal competitors. However, genetic competence must supply some benefit to the *S. mutans* population as ComRS and competence genes are highly conserved across 100 s of sequenced *S. mutans* isolates [45, 46]. The high degree of conservation of ComRS may be associated with how this peptide signaling circuit is integrated with numerous stress response systems [60]. While we have focused extensively on the output effects of peptide signaling, our documented GEP to competitors may impact other aspects of *S. mutans* virulence or fitness not extensively explored here. One example is the upregulation of glucosyltransferases *gtfB* and *gtfC* (higher fold change in *gtfC* compared to *gtfB*), which encode the exoenzymes required for water-insoluble exopolysaccharide production during sucrose-dependent biofilm formation that would impact *S. mutans* microcolony development and spatial organization. Upregulation of *S. mutans gtfC* during growth in multispecies biofilms has been previously reported [50] and corroborates our data here. It is possible that the global transcriptomic response leads to modification of traits, such as acid production and acid tolerance, bacterial adherence, production of secreted molecules, and antimicrobial susceptibility that would modify fitness during competitive interactions. Focusing on the subset of genes and operons that show differential expression in monoculture vs. dual- or multispecies cultivation may highlight new therapeutic targets to study and understand their benefit to the organism. We must also determine how the expression of this select subset of genes is altered as the microbiome profile shifts between caries-free and caries-active states [11]. Importantly, our findings should raise awareness that studying biological systems more closely to their natural setting or in the presence of key environmental factors could dramatically enhance the understanding of how these systems function in vivo to influence health and disease.

## Materials and methods

### Bacterial strains and growth conditions

Strains of *S. mutans* and other *Streptococcus* spp. were cultured in brain heart infusion (BHI) broth (Difco) or CDM [38, 39] supplemented with 20 mM glucose (planktonic plate reader experiments) or 20 mM glucose and 5 mM sucrose (biofilms for microscopy imaging and flow cytometry analysis). Streptococci were grown in a 5% CO_2_ aerobic environment at 37 °C, unless stated otherwise. Antibiotics were added to growth media only during overnight growth in BHI at 1 mg/mL for both kanamycin and spectinomycin and 10 µg/mL for erythromycin. Strains and plasmids are listed in Supplementary Tables 5 and 6, respectively. Cloning strategy and primers used for the *opp* defective strains are listed in Supplementary Table 7.

### Microtiter plate assays

For monitoring of cell density (optical density at 600 nm, OD_600_) and GFP fluorescence (relative fluorescent units, RFUs) over time, *S. mutans* reporter strains and respective cocultures of streptococci were processed as follows. Strains were cultured to an OD_600_ = 0.5 and then each diluted 1:100 into CDM (1:50 combined dilution) at a 1:1 inoculation ratio unless otherwise noted. In quadruplicates, cultures (0.2 mL) were placed in dark-sided, clear bottom, 96-well microtiter plates (Corning). For each fluorescent reporter strain, a control was used that harbored the plasmid without the *gfp* gene to allow subtraction of background fluorescence of cells and/or media components. A mineral oil overlay was added to reduce the effects of oxygen on the bacteria and prevent evaporation. After loading, the 96-well plate was placed in a Synergy HT microtiter plate reader (BioTek). Fluorescence and optical density were measured at 30 min intervals using Gen5 software (BioTek). The settings to measure GFP fluorescence were as follows: excitation at 485 nm, emission at 525 nm; sensitivity 65. Data readings were collected and background fluorescence or OD_600_ were subtracted prior to data visualization using GraphPad Prism 7 (GraphPad Software). See Supplementary information for methods regarding individual experiments.

### CFU determination after microtiter plate assays

After 18 h of monitoring within the Synergy HT microtiter plate reader, an aliquot (0.1 mL) of individual cocultures was removed and added to 0.9 mL of phosphate-buffered saline pH 7.0 (PBS). The cell suspensions were then sonicated four times for 30 s in a sonicating water bath with resting in between on ice to de-chain cells. The cell suspensions were then serially diluted onto BHI agar with or without antibiotic selection to enumerate reporter cells or total cells, respectively. After 48 h incubation at 37 °C in a 5% CO_2_ aerobic atmosphere, CFUs were recorded. The percentage of each strain was determined by subtracting the reporter colonies (selective agar) from a total colony count (nonselective agar). Experiments were repeated at least three times.

### Confocal imaging of biofilm populations

Strains of interest were grown to mid-exponential phase (OD_600_ = 0.5). A 1/100 dilution of each strain was added to 1 mL of CDM supplemented with 20 mM glucose and 7.5 mM sucrose, and 350 μL of these mixtures was used to inoculate one chamber of an ibidi µ-Slide eight-well chamber slide (ibidi GmbH; catalog number, 80826). The samples were incubated at 37 °C in a 5% CO_2_ aerobic atmosphere for 24 h, and 1 µL of blue-fluorescent SYTO® dye 42 nucleic acid stain (Invitrogen^™^, total bacteria) was added to each well and incubated for 15 min at room temperature prior to imaging. Images were acquired using a Nikon Ti2 confocal microscope and Nikon C2plus camera equipped with a Plan Apo *λ* ×60 Oil Objective. For image capture, the following configuration was used: Syto45/DNA (ex 405, em 447, PMT HV 80), EGFP (ex 488, em 510, PMT HV 80), and DsRed2 (ex 561, em 785, PMT HV 110). All z-sections were collected at 1 µm intervals within a 212 × 212 µm field of view at 0.5 s scanning speed. At least five images were acquired from different parts of each biofilm and used for image analysis. Images shown are 3D volume views using alpha blending at an *XY*-viewing plane.

### Flow cytometry

Cocultured biofilms formed with strains inoculated in a 1:1 ratio were grown for 24 h, harvested, washed, and resuspended in 1x PBS. Cells in 5 mL polystyrene round-bottom tubes were sonicated in a water bath for four intervals of 30 s, with resting on ice. Samples were analyzed using an LSR II™ (BD Biosciences) flow cytometer. Forward and side scatter signals were set stringently to allow acquisition of single cells. In total, 5 × 10^4^ cells were counted from each event, at a maximum rate of 2 × 10^3^ cells per second, and each experiment was performed in triplicate. Data were acquired for unstained cells and single-color positive controls so that data collection parameters and compensation could be properly set. The data were collected using FACSDiva (BD Biosciences) and analyzed with FCS Express 6 (De Novo Software). Gating for quadrant analysis was selected by using a dot density plot with forward and side scatter, with gates set to capture the densest section of the plot. *x*- and *y*-axis data represent logarithmic scales of fluorescent intensity (arbitrary units).

### Transcriptome analysis by RNA-Seq

*S. mutans* UA159 and *S.* sp. A12 were grown overnight in CDM. The next day, cultures were harvested by centrifugation and spent supernatant fluid removed into a new vial. Supernatant fluids were treated by adjusting the pH from ~6.3 to 7.0 using 6 N sodium hydroxide and adding 1 M glucose to a final concentration of 20 mM additional glucose. Prior to inoculation, supernates were filter sterilized with a Millex®GP 0.22 µm filter unit containing a Millipore Express® polyethersulfone membrane. *S. mutans* UA159 was inoculated into supernatant fluids, as well as into direct cocultures with *S.* sp. A12 in fresh CDM medium at a 1:50 final dilution ratio (1:100 dilution of each at OD_600 nm_ = 0.5, same as microtiter plate assays). All cultures were grown to OD_600 nm_ = 0.5 before harvesting. RNA extraction, rRNA removal, library construction, and read analysis were conducted as previously described [54, 55, 61]. Briefly, 10 µg of high-quality total RNA was processed using the MICROB*Express*^TM^ Bacterial mRNA Enrichment Kit (Ambion of Life Technologies, Grand Island, NY), twice, before ethanol precipitation and resuspension in 25 µL of nuclease-free water. The quality of enriched mRNA samples was assessed with an Agilent Bioanalyzer (Agilent Technologies, Santa Clara, CA). cDNA libraries were generated from the enriched mRNA samples using the NEBNext® Ultra Directional RNA Library Prep Kit for Illumina (Illumina, San Diego, CA), following instructions from the supplier. Deep sequencing was performed at the University of Florida ICBR facility (Gainesville, FL). Approximately 15 million short reads were obtained for each sample. After removing adapter sequences from each short-read and trimming of the 3′-ends by quality scores [62], the resulting sequences were mapped onto the reference genome of strain UA159 (GenBank Accession No. AE014133) using the short-read aligner. Mapped short-read alignments were then converted into readable formats using SAMTOOLS [63]. For viewing of the mapped reads aligned to the genome, “.bam” files were uploaded into the Integrative Genomics Viewer (version 2.3.55) [64]. A “.csv” file containing raw read counts for each replicate (three) was then uploaded to Degust (http://degust.erc.monash.edu/) and edgeR analysis performed to determine Log2 fold change and false discovery rates (FDR). The *p* value was obtained by taking the −log10 of the FDR. The data files used in this study are available from NCBI-GEO (Gene Expression Omnibus) under Accession No. GSE147421.

### qRT-PCR of cocultures

Cocultures in CDM medium supplemented with 20 mM glucose from a 1:100 initial inoculum of each respective species (1:50 total inoculum) were harvested at OD_600_ = 0.5. Cell lysis was achieved through mechanical disruption (bead beating) and RNA was extracted by acidic phenol phase separation. The RNA was further purified with a RNeasy minikit (QIAGEN) according to the provided protocol and in-column digestion with DNase 1 (QIAGEN). Purified RNA (1 μg) was used to generate cDNA from gene‐specific primers (Supplementary Table 8) using the Superscript III first‐strand synthesis (Invitrogen) reverse transcription protocol. Real‐time PCRs were carried out using an iCyclerQ real‐time PCR detection system (Bio‐Rad) and iQSYBR green supermix (Bio‐Rad) according to the supplier’s protocol. The expression of SMU.996 and SMU.1616c, two unique core genes of *S. mutans*, was used as an internal reference to correct for the amount of total *S. mutans* transcripts present at the time of harvest for each coculture (see Supplementary Table 4).

## Supplementary information

Kaspar 2020 Supplemental Material

Supplemental Movie 1
