# Supplementary material for: Direct interactions with commensal streptococci modify intercellular communication behaviors of Streptococcus mutans
Source: ISME J. 2020 Sep 30;15(2):473–88. doi: 10.1038/s41396-020-00789-7 (PMC8027600; doi:10.1038/s41396-020-00789-7)
Supplement: Supplementary file 1 — Kaspar 2020 Supplemental Material [file 41396_2020_789_MOESM1_ESM.pdf]

Supplemental Material

Direct Interactions with Commensal Streptococci Modify Intercellular Communication Behaviors of *Streptococcus mutans*

Justin R. Kaspar[1,2,#], Kyulim Lee[1], Brook Richard[1], Alejandro R. Walker[1], and Robert A. Burne[1]

[1] Department of Oral Biology, College of Dentistry, University of Florida, Gainesville, Florida

[2] Division of Biosciences, College of Dentistry, Ohio State University, Columbus, Ohio

Running title: Contact-dependent interspecies antagonism

Keywords: microbial interactions, biofilms, cell signaling, genetic competence

[#] Corresponding author

E-mail: Kaspar.17@osu.edu

Table of Contents:

Supplemental Material and Methods: 2

Supplemental Tables: 4

Supplemental Figures: 18

Supplemental References: 30

SUPPLEMENTAL MATERIAL AND METHODS

Microtiter Plate Assays (Expanded)

Figure 2C: 5 µM of synthetic XIP was added to CDM prior to strain inoculation to determine if exogenously added XIP could overcome ComRS signaling inhibition by competitor streptococci. Synthetic XIP (sXIP, aa sequence = GLDWWSL), corresponding to residues 11-17 of ComS, was synthesized and purified to 96% homogeneity by NeoBioSci (Cambridge, MA). The lyophilized sXIP was reconstituted with 99.7% dimethyl sulfoxide (DMSO) to a final concentration of 2 mM and stored in 100 µL aliquots at -20°C.

Figure 5A: Selected strains were grown overnight in BHI with appropriate antibiotics (37°C 5% CO_2). Next morning, cells were harvested by centrifugation, washed and diluted 1:40 into fresh CDM. Cells were incubated for 5 h (till $OD_{600\ nm}$ = 0.5). For thermal deactivation, cells (1 mL total volume in a microcentrifuge tube) were placed at 80°C for 0.5 h in a heating block. After, treated cells were inoculated directly to begin the experiment.

Figure 5B: Two sets of cells were cultured as in Figure 5A: (1) those to be inoculated without treatment for controls, and those to be treated with 4% paraformaldehyde. For paraformaldehyde treatment, cells were harvested by centrifugation and washed with 1X PBS. Cells were then resuspended in 4% paraformaldehyde PBS and kept on the benchtop to incubate for 1 h. After, cells were centrifuged, washed in CDM and resuspended in fresh CDM for inoculation to begin plate reader experiment.

Figure 5C: For dilution series, 20 µL of *S. sp. A12* was inoculated into 2 mL of fresh CDM after reaching mid-exponential growth phase (original 50% in Figure 5C). After vortexing to mix, 1 mL was removed and added to 1 mL of fresh CDM for a 1:2 serial dilution (25% *S. sp. A12*). This process was continued seven additional times to complete the *S. sp. A12* dilution series. 0% represented no *S. sp. A12* addition (same as fresh CDM). After all dilutions were completed, 10 µL of either *S. mutans* UA159 (fluorescent background subtraction) or *S. mutans* P*comX*::*gfp* reporter strain was added, mixed and then plated in the 96 well plate to begin the experiment.

Figure 5D-5F: These figures represent data from one experiment. Either *S. mutans* UA159 (fluorescent background subtraction) or *S. mutans* P*comX*::*gfp* reporter strain were added to 1 mL of CDM and plated into a 96 well plate to begin the experiment. *S. mutans* was inoculated at different ratios so that tested cultures would enter mid-exponential growth at different time points and thus autoactivation of ComRS signaling would initiate separate from each other. 20 µL of *S. mutans* reporter strain was added to 1 mL of CDM in panel D, 15 µL in panel E and 10 µL in panel F. Simultaneously, cultures of either *S. mutans* UA159 or *S. gordonii* DL1 were inoculated 1:100 and incubated at 37˚C and 5% CO_2 atmosphere at the beginning of the experiment. Data from the Synergy plate reader was then closely monitored. At a selected time point when competence activation could be fully detected over background in the higher inoculation culture and beginning to be detected in the middle inoculation cultures (= 4.5 h), the experimental protocol on the plate reader was paused, the 96-well plate carefully removed and 10 µL of either *S. mutans* UA159 or *S. gordonii* DL1 was added to their respective wells. The 96-well plate was then returned to the plate reader and protocol continued to run till completion. See supplemental figure 7.

Figure 6A: Selected competitor strains were grown overnight in BHI with appropriate antibiotics. Next day, cells were harvested by centrifugation, washed and diluted 1:100 into fresh CDM. Cultures were incubated overnight (37°C and 5% CO_2). The next day, cultures were harvested

by centrifugation and spent supernatant fluid moved into a conical tube. Supernates were treated by adjusting pH from ~6.3 to 7.0 using 6N sodium hydroxide and adding back 20 mM of glucose as a carbohydrate source. Supernatants were filter sterilized with a Millex®GP 0.22 µm filter unit containing a Millipore Express® polyethersulfone (PES) membrane prior to reporter inoculation. 10 µL of either *S. mutans* UA159 (fluorescent background subtraction) or *S. mutans* P*comX*::*gfp* reporter strain were added to 1 mL of treated overnight supernatant fluid, mixed and then plated in the 96 well plate to begin the experiment.

Figure 6B: Selected strains were grown overnight in BHI with appropriate antibiotics. Next morning, cells were harvested by centrifugation, washed and diluted 1:40 into fresh CDM. Cells were incubated for 5 h (till $OD_{600\ nm}$ = 0.5). 10 µL of either *S. mutans* UA159 (fluorescent background subtraction) or *S. mutans* P*comX*::*gfp* reporter strain were added to 1 mL of fresh CDM, mixed and then 0.1 mL was plated into the dark-sided, clear bottom 96-well plate. An HTS Transwell®-96 Well insert (Corning) that contains a 0.4 µm polycarbonate membrane was then placed over the pipetted cultures. 0.1 mL of CDM that contained a 1/100 dilution of the competitor species was then added on top of the Transwell insert. After a mineral oil overlay was added, the 96-well plate containing the Transwell insert was then placed into the Synergy2 plate reader to begin the experiment.

Figure 8B: After all inoculated cultures were added to two different 96-well plates, similar to previous experiments but with no mineral oil overlay, the plates were either placed (1) within a 37°C and 5% CO2 incubator or (2) within a GasPak™ EZ Anaerobe Container System with Indicator (Becton, Dickinson and Company), and placed in a regular 37˚C incubator. After 12 h, plates were removed, and data recorded by the Synergy2 plate reader.

<u>Construction of ∆*oppA* Strains</u>

DL1: The *oppA* deletion strain of *S. gordonii* DL1 was constructed using a PCR ligation mutagenesis approach as previously described (1). Briefly, 700 bp fragments both upstream and downstream of the gene of interest were amplified with designed primers and contained a BamHI restriction site in the region closest to the gene. After restriction digest, a non-polar kanamycin resistant cassette was ligated within the arms. This ligated fragment was then transformed into *S. gordonii* DL1 with selection on BHI agar containing 1 mg mL^{-1} kanamycin. Successful replacement was confirmed both by size selection colony PCR and Sanger sequencing (Eurofins Genomics, Louisville, KY, USA). Restriction and DNA-modifying enzymes were obtained from New England Biolabs. PCRs were carried out with 100 ng of chromosomal DNA by using *Taq* DNA polymerase, and PCR products were purified with the QIAquick kit (QIAGEN).

A12: The *oppA* deletion strain of A12 was constructed following methods previously described (2). Briefly, gene disruption was achieved via double-crossover recombination method using linear DNA assembled through Gibson Assembly (New England BioLabs, Beverly, MA). Two DNA fragments flanking the coding sequence of *oppA* were amplified using indicated primer sets. Selected primer sets also contained at least 25 bases of sequence that overlapped with 5' and 3' ends of a nonpolar kanamycin resistance cassette in pALH124. The two flanking DNA fragments and the kanamycin resistance cassette were mixed in equimolar concentrations in a single isothermal ligation reaction. For transformation of A12, overnight cultures of A12 were inoculated into fresh BHI medium and 0.5 ug of prepared DNA was used to transform A12 with 50 nM of A12 sCSP (2, 3) to induce competence. After 5 h of incubation, cells were plated onto BHI agar plates containing kanamycin (1 mg ml^{-1}) and isolated colonies were picked for PCR verification. PCR products from positive colonies were sent for sequence verification.

SUPPLEMENTAL TABLES

Supplemental Table 1: Flow Cytometry Data Analysis

Condition	Sample		Total Events	DAPI+	DsRed+	DsRed+/DAPI+	Average	DsRed-	GFP+	GFP+/DsRed-	Average
**Planktonic**	*S. mutans*	1	47749	30572	10364	33.90%	33.54%	20005	20542	102.68%	102.57%
		2	48940	29331	9857	33.61%		19293	19799	102.62%	
		3	47409	33302	11025	33.11%		22032	22562	102.41%	
	S. gordonii	1	48650	17514	245	1.40%	28.24%	17214	1	0.01%	0.01%
		2	46389	21729	9469	43.58%		10439	1	0.01%	
		3	45763	23024	9149	39.74%		12059	1	0.01%	
	S. sp. A12	1	44582	18440	4622	25.07%	26.58%	13732	0	0.00%	0.01%
		2	45959	16580	4780	28.83%		11720	2	0.02%	
		3	46798	17957	4640	25.84%		13227	0	0.00%	
**Biofilm**	*S. mutans*	1	46424	27129	14275	52.62%	52.16%	12627	13672	108.28%	107.15%
		2	46619	26454	13785	52.11%		12470	13374	107.25%	
		3	47274	26513	13724	51.76%		12556	13300	105.93%	
	S. gordonii	1	47276	16999	5432	31.95%	29.84%	9852	2	0.02%	0.01%
		2	46713	15510	4648	29.97%		9401	1	0.01%	
		3	46393	18463	5094	27.59%		11788	0	0.00%	
	S. sp. A12	1	45871	11781	2170	18.42%	23.22%	9547	9	0.09%	0.04%
		2	45764	12873	3969	30.83%		8783	1	0.01%	
		3	45928	13372	2731	20.42%		10566	3	0.03%	

Supplemental Table 2. Differential gene expression between *S. mutans* UA159 grown in *S. sp.* A12 spent supernatant fluid compared to growth in its own spent supernatant fluid. Only significant genes (≥(-)1.5 Log2 fold change and ≥ 4 -log10 P-values) are shown.

Geneid	Gene Name	Gene Fuction Lookup	Log2 Fold Change	Pvalue - log10
SMU.166	NA	hypothetical protein	2.95	14.58
SMU.167	NA	hypothetical protein	2.74	15.54
SMU.168	NA	transcriptional regulator	2.67	15.35
SMU.1302	adcA lraI sloC	zinc ABC transporter, substrate-binding protein	2.59	9.01
SMU.1301c	NA	methyltransferase related segment	2.35	9.13
SMU.1995c	adcR zitR	zinc transport transcriptional repressor	2.29	7.72
SMU.1994	adcC	zinc ABC transport, ATP-binding protein	2.19	9.27
SMU.1300c	NA	conserved hypothetical protein	2.16	8.10
SMU.1993	adcB	zinc ABC transporter, permease	2.08	8.64
SMU.1299c	NA	methyltransferase, possible acetate kinase	1.99	7.48
SMU.299c	ip	bacteriocin peptide precursor	1.72	10.94
SMU.1165c	acrR	transcriptional regulator (TetR/AcrR family)	1.67	10.47
SMU.992	NA	hypothetical protein	1.60	12.33
SMU.1098c	yogA	oxidoreductase	1.56	11.67
SMU.1939c	atmD	amino acid ABC transport ATP-binding protein	-1.51	10.20
SMU.932	NA	conserved hypothetical protein	-1.51	6.33
SMU.113	pfk	fructose-1-phosphate kinase	-1.54	4.92
SMU.510c	NA	deoxyribonuclease	-1.56	8.63
SMU.1938c	atmE	amino acid ABC transport permease	-1.57	9.08
SMU.933	atmA	amino acid ABC transporter, amino acid substrate-binding protein	-1.60	5.55
SMU.115	NA	PTS system, fructose-specific IIA component	-1.61	4.60
SMU.935	NA	amino acid ABC transporter, permease protein	-1.62	5.40
SMU.1937	cnhA	hydrolase, carbon-nitrogen family	-1.63	10.80
SMU.1803c	NA	conserved hypothetical protein	-1.69	6.09
SMU.934	NA	amino acid ABC transporter, permease protein	-1.78	8.45
SMU.1674	patB	aminotransferase class II	-1.80	14.40
SMU.1520	glnH	amino acid ABC transporter, substrate-binding protein	-1.82	12.11
SMU.114	NA	PTS system, fructose-specific IIBC component	-1.84	8.15
SMU.1487	NA	conserved hypothetical protein	-1.98	10.33
SMU.1522	glnP	amino acid (glutamine) ABC transporter permease	-1.98	14.83
SMU.563	arcB argF	ornithine carbamoyltransferase	-2.00	14.80
SMU.1521	glnM	amino acid (glutamine) ABC transporter permease	-2.02	10.78
SMU.116	lacD	tagatose 1,6-aldolase	-2.07	7.37
SMU.149	tpn	transposase fragment (IS605/IS200-like)	-2.09	7.17

SMU.335	argH	argininosuccinate lyase	-2.17	16.19
SMU.334	argG	argininosuccinate synthase	-2.34	14.72
SMU.873	metC metE	5-methyltetrahydropteroyltriglutamate--homocysteine S-methyltransferase	-2.37	12.66
SMU.364	glnA	glutamine synthetase type 1	-2.39	15.23
SMU.1405c	NA	conserved hypothetical protein	-2.45	11.77
SMU.1404c	NA	conserved hypothetical protein	-2.49	13.88
SMU.365	gltA gltB	glutamate synthase, large subunit	-2.49	13.18
SMU.366	gltD	glutamate synthase, small subunit	-2.49	13.08
SMU.1402c	NA	conserved hypothetical protein	-2.51	14.46
SMU.874	metH	5-methyltetrahydrofolate--homocysteine methyltransferase	-2.63	12.87
SMU.1403c	NA	conserved hypothetical protein	-2.76	13.74
SMU.363	glnR	transcriptional regulator; glutamine synthetase repressor	-2.80	15.16
SMU.1958c	levF	fructose-specific Enzyme IIC component	-2.81	4.54
SMU.984	NA	hypothetical protein	-2.92	10.06
SMU.1957	levG ptnD	fructose-specific Enzyme IID component	-2.94	4.32
SMU.1956c	NA	conserved hypothetical protein	-2.96	4.19
SMU.503c	NA	hypothetical protein	-3.05	10.09
SMU.214c	NA	hypothetical protein	-3.06	14.48
SMU.663	argC	N-acetyl-gamma-glutamyl-phosphate reductase	-3.10	15.09
SMU.204c	NA	hypothetical protein	-3.19	14.65
SMU.217c	NA	conserved hypothetical protein; Streptococcus-specific protein	-3.20	14.17
SMU.199c	NA	hypothetical protein	-3.21	16.37
SMU.666	argD	N-acetylornithine aminotransferase	-3.24	15.67
SMU.216c	NA	hypothetical protein	-3.26	14.54
SMU.215c	NA	hypothetical protein	-3.27	13.50
SMU.665	argB	acetylglutamate kinase	-3.27	13.54
SMU.213c	NA	hypothetical protein	-3.28	13.92
SMU.2057c	cadA cadD	cadmium-efflux ATPase, E1-E2 (heavy metal-transporting ATPase)	-3.29	5.89
SMU.210c	NA	hypothetical protein	-3.31	16.45
SMU.664	argJ	ornithine acetyltransferase / N-acetylglutamate synthase	-3.33	15.49
SMU.198c	tpn	conjugative transposon protein	-3.34	16.01
SMU.1658	nrgA	ammonium transporter, NrgA protein	-3.35	14.57
SMU.201c	NA	conserved hypothetical protein	-3.35	14.96
SMU.209c	NA	hypothetical protein	-3.36	15.82
SMU.207c	NA	transcriptional regulator	-3.37	17.14
SMU.208c	NA	conserved hypothetical protein, FtsK/SpoIIIE family	-3.37	15.73
SMU.193c	NA	conserved hypothetical protein	-3.37	16.67
SMU.202c	NA	conserved hypothetical protein/Streptococcus-specific protein	-3.38	16.22
SMU.195c	NA	hypothetical protein	-3.38	17.33
SMU.197c	NA	hypothetical protein	-3.40	16.17
SMU.196c	NA	immunogenic secreted protein (transfer protein)	-3.43	16.11
SMU.205c	NA	conserved hypothetical protein	-3.44	15.46

SMU.212c	NA	hypothetical protein	-3.45	13.44
SMU.206c	NA	hypothetical protein	-3.46	18.61
SMU.200c	NA	hypothetical protein	-3.48	16.77
SMU.211c	NA	hypothetical protein	-3.49	15.38
SMU.194c	NA	conserved hypothetical protein, phage-related	-3.54	14.88
SMU.191c	NA	phage-related integrase	-3.55	16.86
SMU.1657c	glnB	nitrogen regulatory protein PII	-3.77	13.23
SMU.673	NA	conserved hypothetical protein	-3.92	17.23
SMU.609	bsp	cell wall protein precursor	-4.24	17.47
SMU.670	acnA citB	aconitate hydratase; aconitase A	-4.50	19.03
SMU.672	citC icd	isocitrate dehydrogenase	-4.70	17.84
SMU.671	citZ	citrate synthase	-4.73	18.66

Supplemental Table 3. Differential gene expression between direct coculture of *S. mutans* UA159 with *S. sp.* A12 compared to growth in *S. sp. A12* spent supernatant fluid. Only significant genes ($\geq$(-)1.5 Log2 fold change and $\geq$ 4 -log10 P-values) are shown.

Geneid	Gene Name	Gene Fuction Lookup	Log2 Fold Change	Pvalue - log10
SMU.63c	NA	conserved hypothetical protein	3.73	16.88
SMU.1764c	NA	conserved hypothetical protein	3.36	16.92
SMU.1763c	NA	conserved hypothetical protein	3.19	18.98
SMU.1761c	NA	conserved hypothetical protein	3.12	14.63
SMU.1760c	NA	conserved hypothetical protein	3.10	18.83
SMU.1804c	NA	hypothetical protein	3.10	9.30
SMU.1762c	NA	conserved hypothetical protein	3.08	13.39
SMU.1758c	NA	conserved hypothetical protein	2.86	17.31
SMU.1757c	NA	conserved hypothetical protein	2.81	15.40
SMU.1754c	NA	conserved hypothetical protein	2.70	15.68
SMU.1755c	NA	conserved hypothetical protein	2.67	13.33
SMU.1753c	NA	conserved hypothetical protein	2.64	15.79
SMU.510c	NA	deoxyribonuclease	2.50	12.07
SMU.503c	NA	hypothetical protein	2.34	7.50
SMU.1803c	NA	conserved hypothetical protein	2.16	7.43
SMU.984	NA	hypothetical protein	2.09	6.92
SMU.1752c	NA	hypothetical protein	1.91	9.40
SMU.1750c	NA	hypothetical protein	1.86	11.58
SMU.502	NA	conserved hypothetical protein	1.82	13.11
SMU.1005	gtfC	glucosyltransferase-SI	1.81	14.56
SMU.501	NA	hypothetical protein	1.76	11.51
SMU.1403c	NA	conserved hypothetical protein	1.75	9.31
SMU.1879	manN ptnD	mannose PTS system component IID	1.75	10.27
SMU.1878	manM ptnC	mannose PTS system component IIC	1.71	8.86
SMU.609	bsp	cell wall protein precursor	1.69	9.52
SMU.1877	manL ptnAB	mannose PTS system component IIAB	1.68	8.15
SMU.1402c	NA	conserved hypothetical protein	1.61	10.09
SMU.130	acoL adhD	dihydrolipoamide dehydrogenase	-1.50	5.26
SMU.1603	gloA lguL	lactoylglutathione lyase	-1.50	10.85
SMU.1963c	levQ	sugar-binding periplasmic protein	-1.51	9.02
SMU.1091	wapE	hypothetical protein (possible cell wall protein)	-1.51	13.56
SMU.329	NA	conserved hypothetical protein	-1.52	12.49
SMU.725c	NA	conserved hypothetical protein	-1.52	9.52
SMU.1194	yurY	ABC transporter, ATP-binding protein	-1.53	8.89
SMU.2076c	NA	hypothetical protein	-1.54	6.11
SMU.1042	NA	conserved hypothetical protein; possible ABC transporter, permease	-1.57	11.26

SMU.1412c	yhcA	ABC transporter, ATP-binding and permease fusion protein	-1.58	12.53
SMU.1945	NA	conserved hypothetical protein	-1.58	4.41
SMU.665	argB	acetylglutamate kinase	-1.59	4.42
SMU.2028	ftf sacB	fructosyltransferase	-1.60	7.87
SMU.1041	ecsA yfiL	ABC transporter, ATP-binding protein	-1.62	13.66
SMU.1070c	NA	conserved hypothetical protein	-1.62	5.34
SMU.2083c	NA	hypothetical protein	-1.62	9.35
SMU.647	NA	O-methyltransferase	-1.63	12.26
SMU.1964c	levR	two-component response regulator	-1.64	9.68
SMU.67	NA	acyltransferase	-1.64	11.28
SMU.500	yfiA	ribosome-associated protein	-1.64	9.39
SMU.1867c	adhB	alcohol dehydrogenase	-1.66	9.05
SMU.1902c	NA	hypothetical protein	-1.69	8.61
SMU.840c	NA	hypothetical protein	-1.70	7.68
SMU.1869	trxA	thioredoxin	-1.74	8.53
SMU.1047c	NA	hypothetical protein	-1.75	5.68
SMU.353	NA	thiamine pyrophosphokinase	-1.75	13.73
SMU.1243	ltrA	conserved hypothetical protein (possible low temperature requirement protein A)	-1.75	5.73
SMU.664	argJ	ornithine acetyltransferase / N-acetylglutamate synthase	-1.76	6.29
SMU.66	NA	conserved hypothetical protein; possible phosphatidylinositol-4-phosphate 5-kinase	-1.77	12.20
SMU.807	NA	conserved hypothetical protein (possible membrane protein)	-1.83	10.70
SMU.1195	NA	ABC transporter permease protein	-1.83	10.30
SMU.1117	naoX nox nox-2	H2O-forming NADH Oxidase	-1.83	10.91
SMU.354	NA	conserved hypothetical protein	-1.84	14.13
SMU.1116c	NA	hypothetical protein	-1.86	11.07
SMU.356	purR xpt	purine operon repressor/xanthine phosphoribosyltransferase	-1.87	12.62
SMU.1197	NA	conserved hypothetical protein	-1.87	9.04
SMU.352	rpe	ribulose-phosphate-3-epimerase	-1.93	11.18
SMU.355	cbf1 yhaM	CMP-binding factor 1	-1.97	13.51
SMU.2027	NA	transcriptional regulator/repressor	-1.97	12.47
SMU.1965c	levS	histidine kinase	-2.04	11.72
SMU.153	NA	hypothetical protein	-2.06	6.37
SMU.764	ahpC	alkyl hydroperoxide reductase, subunit C	-2.19	11.64
SMU.765	ahpF nox1	alkyl hydroperoxide reductase, subunit F	-2.25	11.26
SMU.2084c	NA	conserved hypothetical protein (possible arsenate reductase)	-2.29	11.06
SMU.299c	ip	bacteriocin peptide precursor	-2.33	12.29
SMU.65	ptp	protein tyrosine-phosphatase	-2.38	9.73
SMU.862	NA	conserved hypothetical protein	-2.38	14.29
SMU.152	NA	hypothetical protein	-2.51	9.16
SMU.423	nlmD	possible bacteriocin	-2.51	6.35
SMU.1993	adcB	zinc ABC transporter, permease	-2.52	9.25
SMU.64	ruvB	Holliday junction DNA helicase	-2.54	12.41

SMU.864	NA	ABC transporter, permease component	-2.57	13.93
SMU.863	NA	ABC transporter, ATP-binding protein	-2.58	14.37
SMU.1995c	adcR zitR	zinc transport transcriptional repressor	-2.58	7.43
SMU.1904c	NA	hypothetical protein	-2.59	9.94
SMU.1903c	NA	hypothetical protein	-2.60	10.54
SMU.151	nlmB	non-lantibiotic mutacin IV B	-2.61	6.24
SMU.150	nlmA	non-lantibiotic mutacin IV A	-2.61	6.20
SMU.1994	adcC	zinc ABC transport, ATP-binding protein	-2.62	9.77
SMU.1908c	NA	hypothetical protein	-2.64	9.08
SMU.1322	butA	acetoin reductase	-2.66	16.42
SMU.507	glcR rdrB	transcriptional regulator, DeoR family	-2.71	13.64
SMU.167	NA	hypothetical protein	-2.72	14.67
SMU.1905c	NA	hypothetical protein	-2.73	9.59
SMU.1299c	NA	methyltransferase, possible acetate kinase	-2.73	8.44
SMU.1966c	rbsB	ABC transport ribose-binding protein, periplasmic	-2.74	13.88
SMU.1302	adcA lraI sloC	zinc ABC transporter, substrate-binding protein	-2.76	9.62
SMU.772	gbpD	bifunctional glucan-binding protein D and lipase	-2.76	14.88
SMU.508	NA	conserved hypothetical protein (possible hydrolase)	-2.77	14.72
SMU.1912c	NA	hypothetical protein	-2.79	10.13
SMU.1910c	NA	hypothetical protein	-2.81	8.25
SMU.1300c	NA	conserved hypothetical protein	-2.87	8.80
SMU.1909c	NA	hypothetical protein	-2.87	8.47
SMU.1906c	NA	bacteriocin-related protein	-2.87	8.05
SMU.168	NA	transcriptional regulator	-2.91	14.75
SMU.2085	recA	recombinase A	-2.94	17.11
SMU.166	NA	hypothetical protein	-2.95	14.03
SMU.1913c	NA	hypothetical protein; immunity protein, BLpL-like	-2.97	9.22
SMU.1055	radC	DNA repair protein RadC	-2.98	15.50
SMU.506	ssuRB	type II restriction endonuclease	-2.98	17.19
SMU.646	gph	hydrolase (possible phosphoglycolate phosphatase)	-2.99	17.43
SMU.838	gor gshR	glutathione reductase	-3.02	15.21
SMU.1301c	NA	methyltransferase related segment	-3.05	10.81
SMU.1914c	bip cipB	bacteriocin protein, BlpO-like	-3.06	6.64
SMU.1003	gidA	glucose-inhibited division protein	-3.09	13.79
SMU.1916	comD	histidine kinase	-3.10	16.12
SMU.627	NA	conserved hypothetical protein	-3.12	15.72
SMU.1917	comE	response regulator	-3.22	15.49
SMU.645	pepB	PepB oligopeptidase	-3.30	16.66
SMU.505	dpn	adenine-specific DNA methylase (DpnIIB)	-3.43	17.40
SMU.2086	cinA	competence damage-inducible protein A	-3.45	19.22
SMU.1002	topA	DNA topoisomerase I	-3.47	13.34
SMU.1997	comX	competence-specific sigma factor	-3.56	13.74

SMU.1978	ackA comYI	acetate kinase	-3.57	11.94
SMU.499	comFC	late competence protein required for DNA uptake	-3.81	14.46
SMU.498	comFA	late competence protein F	-3.95	16.03
SMU.539c	comC hopD	prepilin peptidase type IV	-3.97	12.03
SMU.644	coiA	competence protein CoiA	-4.06	18.00
SMU.836	NA	hypothetical protein	-4.12	15.10
SMU.837	ycgG	oxidoreductase, aldo/keto reductase family	-4.12	16.09
SMU.1967	ssbA	single-stranded DNA-binding protein	-4.36	16.72
SMU.1979c	ythI ytxK	conserved hypothetical protein, methyltransferase domain	-4.41	17.58
SMU.1001	dprA	DNA processing protein, Smf family	-4.42	17.76
SMU.1981c	comG comGF	competence protein G	-4.44	16.78
SMU.1983	comYD	competence protein ComYD	-4.50	16.06
SMU.625	celA comEA	competence protein	-4.55	16.56
SMU.1982c	NA	conserved hypothetical protein	-4.55	17.05
SMU.1980c	NA	conserved hypothetical protein	-4.56	15.95
SMU.1985	comGB comYB	competence protein; general (type II) secretory pathway protein	-4.57	14.47
SMU.1987	comGA comYA	late competence protein; type II secretion system protein E	-4.58	15.43
SMU.1984	comYC	competence protein ComYC	-4.62	16.37
SMU.769	NA	transposase	-4.62	16.37
SMU.626	celB comEC	competence protein; possible integral membrane protein	-4.68	15.11

Supplemental Table 4: Determination of Correction Factors for qRT-PCR Analysis.

Sample	SMU-1	SMU-2	SMU-3
SMU_996 Reads	69931	105908	79381
Average			85074
996 CF			
SMU_1616c Reads	14977	16808	13738
Average			15174
1616 CF			
Correction Factor			
**Sample**	**A12-1**	**A12-2**	**A12-3**
SMU_996 Reads	53346	76730	66760
Average			
996 CF	1.6	1.1	1.3
SMU_1616c Reads	6827	8833	6908
Average			
1616 CF	2.2	1.7	2.2
Correction Factor	**1.9**	**1.4**	**1.7**
**Sample**	**DL-1**	**DL-2**	**DL-3**
SMU_996 Reads	28842	44929	114249
Average			
996 CF	2.9	1.9	0.7
SMU_1616c Reads	4950	6980	12736
Average			
1616 CF	3.1	2.2	1.2
Correction Factor	**3.0**	**2.0**	**1.0**
**Sample**	**SK150-1**	**SK150-2**	**SK150-3**
SMU_996 Reads	28192	46675	33579
Average			
996 CF	3.0	1.8	2.5
SMU_1616c Reads	2881	6987	3582
Average			
1616 CF	5.3	2.2	4.2
Correction Factor	**4.1**	**2.0**	**3.4**

Supplemental Table 5: Strains used in this study.

Species	Strains	Genotype or description	Reference or source
Streptococcus mutans	UA159		Lab stock
	pDL278_P*comS*::*gfp* / UA159	UA159 harboring *gfp* fluorescent reporter of P*comS*	(4)
	pDL278_P*comX*::*gfp* / UA159	UA159 harboring *gfp* fluorescent reporter of P*comX*	(5)
	pDL278_P*comX*::*gfp*, pIB184 / UA159	UA159 harboring *gfp* fluorescent reporter of P*comX* and pIB184 (empty vector)	(5)
	pDL278_P*comX*::*gfp*, pIB184_P23::*comS* / UA159	UA159 harboring *gfp* fluorescent reporter of P*comX* and overexpression of *comS* by P23 promoter	(6)
	Smu52	*S. mutans* isolate	(7, 8)
	pDL278_P*comX*::*gfp* / Smu52	Smu52 harboring *gfp* fluorescent reporter of P*comX*	This study
	Smu63	*S. mutans* isolate	(7, 8)
	pDL278_P*comX*::*gfp* / Smu63	Smu63 harboring *gfp* fluorescent reporter of P*comX*	This study
	Smu86	*S. mutans* isolate	(7, 8)
	pDL278_P*comX*::*gfp* / Smu86	Smu86 harboring *gfp* fluorescent reporter of P*comX*	This study
	Smu89	*S. mutans* isolate	(7, 8)
	pDL278_P*comX*::*gfp* / Smu89	Smu89 harboring *gfp* fluorescent reporter of P*comX*	This study
	Smu93	*S. mutans* isolate	(7, 8)
	pDL278_P*comX*::*gfp* / Smu93	Smu93 harboring *gfp* fluorescent reporter of P*comX*	This study
	Smu94	*S. mutans* isolate	(7, 8)
	pDL278_P*comX*::*gfp* / Smu94	Smu94 harboring *gfp* fluorescent reporter of P*comX*	This study
	Smu101	*S. mutans* isolate	(7, 8)
	pDL278_P*comX*::*gfp* / Smu101	Smu101 harboring *gfp* fluorescent reporter of P*comX*	This study
	Smu105	*S. mutans* isolate	(7, 8)
	pDL278_P*comX*::*gfp* / Smu105	Smu105 harboring *gfp* fluorescent reporter of P*comX*	This study
	Smu107	*S. mutans* isolate	(7, 8)
	pDL278_P*comX*::*gfp* / Smu107	Smu107 harboring *gfp* fluorescent reporter of P*comX*	This study
	Smu108	*S. mutans* isolate	(7, 8)
	pDL278_P*comX*::*gfp* / Smu108	Smu108 harboring *gfp* fluorescent reporter of P*comX*	This study
	Smu109	*S. mutans* isolate	(7, 8)
	pDL278_P*comX*::*gfp* / Smu109	Smu109 harboring *gfp* fluorescent reporter of P*comX*	This study
Streptococcus gordonii	DL1		Lab stock
	DL1 ∆*spxB*	*spxB* (Sgo_0292) :: Non-polar Kanamycin resistance cassette	(9)
	DL1 ∆*oppA*	*oppA* (Sgo_1712) :: Non-polar Kanamycin resistance cassette	This study
	BCC09	Clinical isolate	(10)
	BCC27	Clinical isolate	(10)
	BCC29	Clinical isolate	(10)
	BCC32	Clinical isolate	(10)
	BCC57	Clinical isolate	(10)

	BCC62	Clinical isolate	(10)
	BCA05	Clinical isolate	(10)
	BCA07	Clinical isolate	(10)
	BCA10	Clinical isolate	(10)
	BCA22	Clinical isolate	(10)
Streptococcus sanguinis	SK150		
	BCC03	Clinical isolate	(10)
	BCC04	Clinical isolate	(10)
	BCC14	Clinical isolate	(10)
	BCC23	Clinical isolate	(10)
	BCC37	Clinical isolate	(10)
	BCC39	Clinical isolate	(10)
	BCC46	Clinical isolate	(10)
	BCC54	Clinical isolate	(10)
	BCC61	Clinical isolate	(10)
	BCC64	Clinical isolate	(10)
S. sp. A12 and A12-likes	A12		
	A12 Δ*spxB*	*spxB* (ATM98_05265) :: Non-polar Kanamycin resistance cassette	(2)
	A12 Δ*sgc*	*sgc* (ATM98_08980) :: Erythomycin resistance cassette	(3)
	A12 Δ*pcfO*	*pcfO* (ATM98_08220) :: Non-polar Kanamycin resistance cassette	(2)
	A12 Δ*oppA*	*oppA* (ATM98_08725) :: Non-polar Kanamycin resistance cassette	This study
	A1	Clinical isolate	(2)
	A13	Clinical isolate	(2)
	BCC21	Clinical isolate	(2)
	G1	Clinical isolate	(2)
	G2	Clinical isolate	(2)

Supplemental Table 6: Plasmids used in this study.

Plasmid	Description	Source
pDL278	*Escherichia coli* - *Streptococcus* shuttle vector, spectinomycin resistance carried	(11)
pDL278_P*comX*::*gfp*	P*comX* promoter fused to *gfp* reporter gene between EcoRI and SphI cut sites	(5)
pDL278_P*comS*::*gfp*	P*comS* promoter fused to *gfp* reporter gene between EcoRI and SphI cut sites	(4)
pIB184	Shuttle expression plasmid harboring constitutive P23 promoter, erythromycin resistance carried	(12)
pIB184_P23::*comS*	*comS* inserted behind constitutively active P23 promoter	(13)

Supplemental Table 7: Cloning Primers used in this study.

Primer Name	Sequence	Tm (˚C)	Use
Sgo_1712-A	GGA TTT GAA GCT GGT AGT GG	53	Amplification of 5' arm of Δ*oppA S. gordonii* mutant
Sgo_1712-B	CCT **GGA TCC** ATC GTA TAT TCT CC	54	Amplification of 5' arm of Δ*oppA S. gordonii* mutant
Sgo_1712-C	CTC TAA **GGA TCC** AGA AAG TTC TTT TGG G	57	Amplification of 3' arm of Δ*oppA S. gordonii* mutant
Sgo_1712-D	GGG TAA GAA TTT CCT AAG TTC AAG G	54	Amplification of 3' arm of Δ*oppA S. gordonii* mutant
Sgo_1712-F	GGA AAG AGG ATA ATA AGA CCC G	52	Sequencing of 5' arm of ΔoppA S. gordonii mutant
Sgo_1712-R	ATG GTA TAG GTC AAA GCC G	52	Sequencing of 3' arm of ΔoppA S. gordonii mutant
A12_1729-A	GCGGCGACCAAGATTCTTCGAAACACCTTGG	66	Amplification of 5' arm of Δ*oppA* A12 mutant
A12_1729-B	GCCATTTATTATTTCCTTCCTCTTTTAGCAAAAACTTTACTTTTTTTCATTTTGAG	62	Amplification of 5' arm of Δ*oppA* A12 mutant
A12_1729-C	ATATTTTACTGGATGAATTGTTTTAGTAGACACGTTAAATAATCACCTTCATCA	62	Amplification of 3' arm of Δ*oppA* A12 mutant
A12_1729-D	CTTGTGTAATAACCTGAAGAACCGGCG	60	Amplification of 3' arm of Δ*oppA* A12 mutant
A12_1729-F	GTACATCTTACTATCTAGGCTTC	50	Sequencing of 5' arm of Δ*oppA* A12 mutant
A12_1729-R	GCGGGCAATATCTTGAGCATCTGCC	62	Sequencing of 3' arm of Δ*oppA* A12 mutant

BOLD text notes BamHI cut site within primer sequence (GAA TCC)

UNDERLINED text notes insertion of NP-kanamycin marker sequence within primer.

Supplemental Table 8: qRT-PCR Primers used in this study.

Gene Group	Gene		Primer Sequence (5' - 3')	Tm	Product Size (bp)
**Control**	SMU.996	F	ACTTTATGGCATCGGTCCAG	60	106
		R	ACCAACACATACAGCCGTCA	60	
	SMU.1616c	F	CCTGCGTTTCTTTTCTGAGG	60	101
		R	CCAATGCAAAGCTGTCATCA	61	
**Upregulated**	SMU.63c	F	TCAAAGGGAACCAAACAAGC	60	107
		R	GCCTTTGTTTTTGCAACGAT	60	
	SMU.501	F	GCAGAGGCTTTAGCCATAGG	59	91
		R	CTTCAGCAACCGTGTCGTTA	59	
	SMU.609	F	CAATTGCCAGCAAGTGAAAA	60	94
		R	TTGTTTGAGCTGCTGCCTTA	60	
	SMU.1005	F	GGCTGCGTCAGACTATTTCC	60	103
		R	TAAAGCAATGCCCCTTTTTG	60	
	SMU.1764c	F	ATTTTTGCTGGGCATGTTTC	60	107
		R	TAGCACCAGCATAGGCATGA	60	
	SMU.1877	F	AAATTGCAGCCTCAGGAAGA	60	95
		R	TTGCCATCACCAATGACAGT	60	
**Downregulated**	SMU.863	F	TGGTAACGCATGAACCTGAG	60	90
		R	CCATTTGCTGGCTGTCTTCT	60	
	SMU.1302	F	AGGATAAAGGGGCTGGAGAA	60	92
		R	GCCCCAGAAAAGATGGAAGT	60	
	SMU.1914c	F	TTGTGCAGCAGGTATTGCTC	60	100
		R	AAAGGCACCAGTGCCAATAG	60	
	SMU.1917	F	AGCCCATAAGCTCTGCCTTT	60	104
		R	AGCGATGGCACTGAAAAAGT	60	
	SMU.1994	F	AAGTCTTTCTGGCGGACAAA	60	105
		R	CCTGTATCCATTCCCGTTGT	60	
	SMU.1997	F	GGTTAAGCCAATTGTATGGAAA	58	96
		R	TGGTGCAAAATCAACATTCC	60	

SUPPLEMENTAL FIGURES AND FIGURE LEGENDS

Supplemental Figure 1

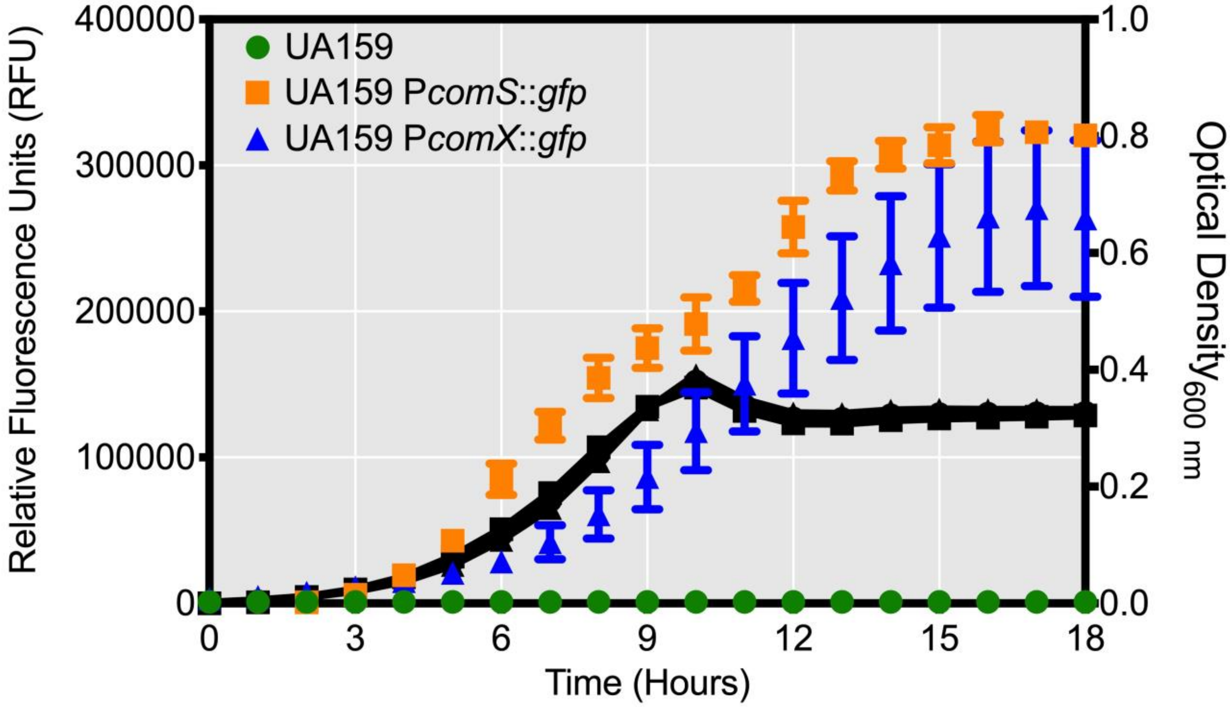

Autoactivation of ComRS signaling in CDM medium. *Streptococcus mutans* strain UA159 harboring plasmid pDL278 that contained a copy of the *gfp* gene under the control of either the P*comS* (orange circles) or P*comX* (blue squares) promoter was grown in CDM medium to mid-exponential log phase ($OD_{600\ nm}$ = 0.5). Cultures were then back diluted 1/50 in CDM and monitored by both fluorescence signal and optical density for 18 h in a Synergy2 multimode plate reader (BioTek). The data shows that ComRS signaling spontaneously activates when either reporter strain is grown in CDM medium and is cell density dependent. Recorded relative fluorescent units (RFUs) are shown in unconnected, colored symbols and plotted on the left y-axis while optical density ($OD_{600\ nm}$) over the course of the experiment is plotted in black, connected lines. The experiment was performed with three biological replicates recorded in technical quadruplets.

Supplemental Figure 2

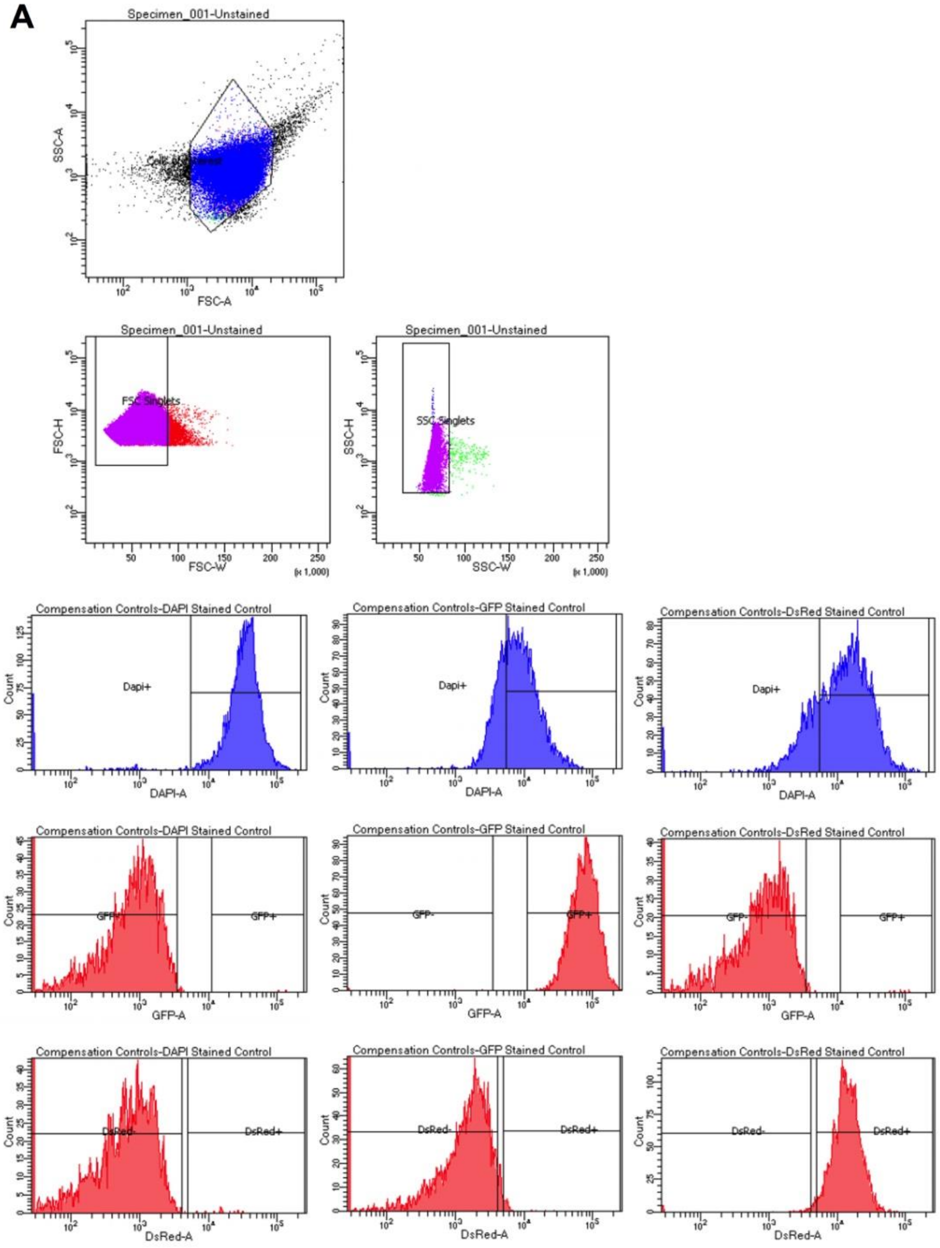

Set-up of Gating for Flow Cytometry Experiment. (A) Top: Forward and side scattering plots for determination of cell size during flow cytometry experiments. Gates were set up to select for single cells (singlets) for further analysis over doublets and cell aggregates. Below: Selection of gates with control cultures prior to analysis of cocultures. All cells were gated on DAPI+ signal with a DAPI (only) stained control (left column). Then, individual cultures producing only GFP (middle) or DsRed (right) were used to set gates within histograms to determine cell counts of either GFP+ or DsRed+ of the cocultures.

B

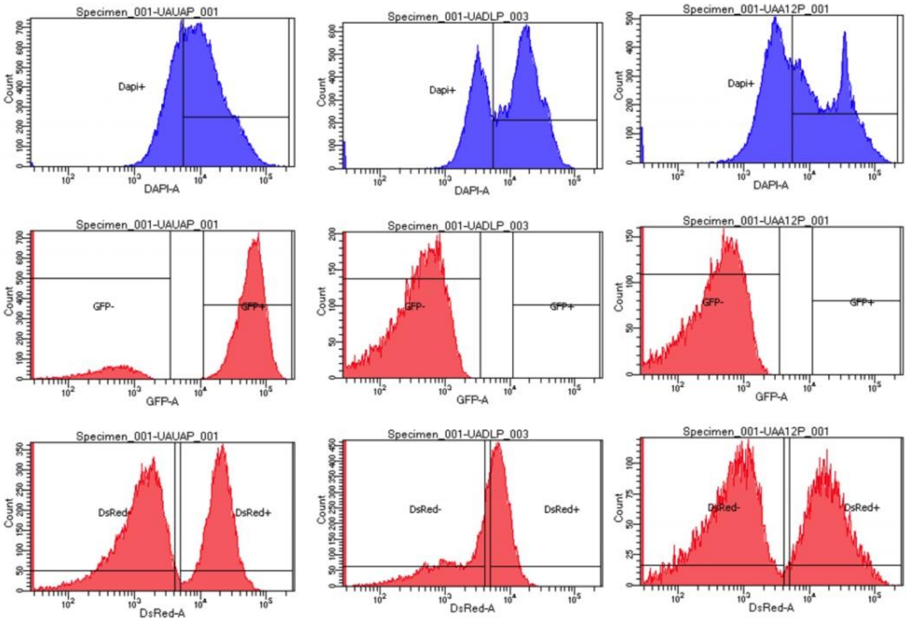

C

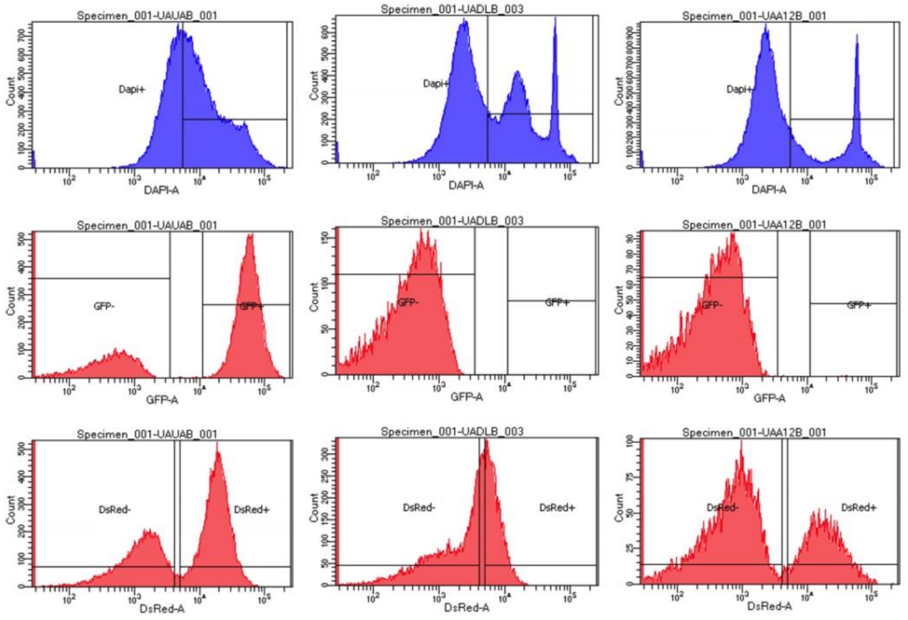

Representative plots from Flow Cytometry Coculture Experiments. (B) Cocultures of *S. mutans* P*comX*::*gfp* reporter with either *S. mutans* UA159 (left column), *S. gordonii* DL1 (middle) or *S. sp. A12* (right) containing a constitutive *dsRed2* plasmid (pDL278_P23::*dsRed2*) grown in planktonic culture for 24 h. Graphs of one replicate show results of DAPI staining, GFP or DsRed production. (C) Cocultures of *S. mutans* P*comX*::*gfp* reporter with either *S. mutans* UA159 (left column), *S. gordonii* DL1 (middle) or *S. sp. A12* (right) containing a constitutive *dsRed* plasmid (pDL278_P23::*dsRed*) grown in biofilms for 24 h.

Supplemental Figure 3

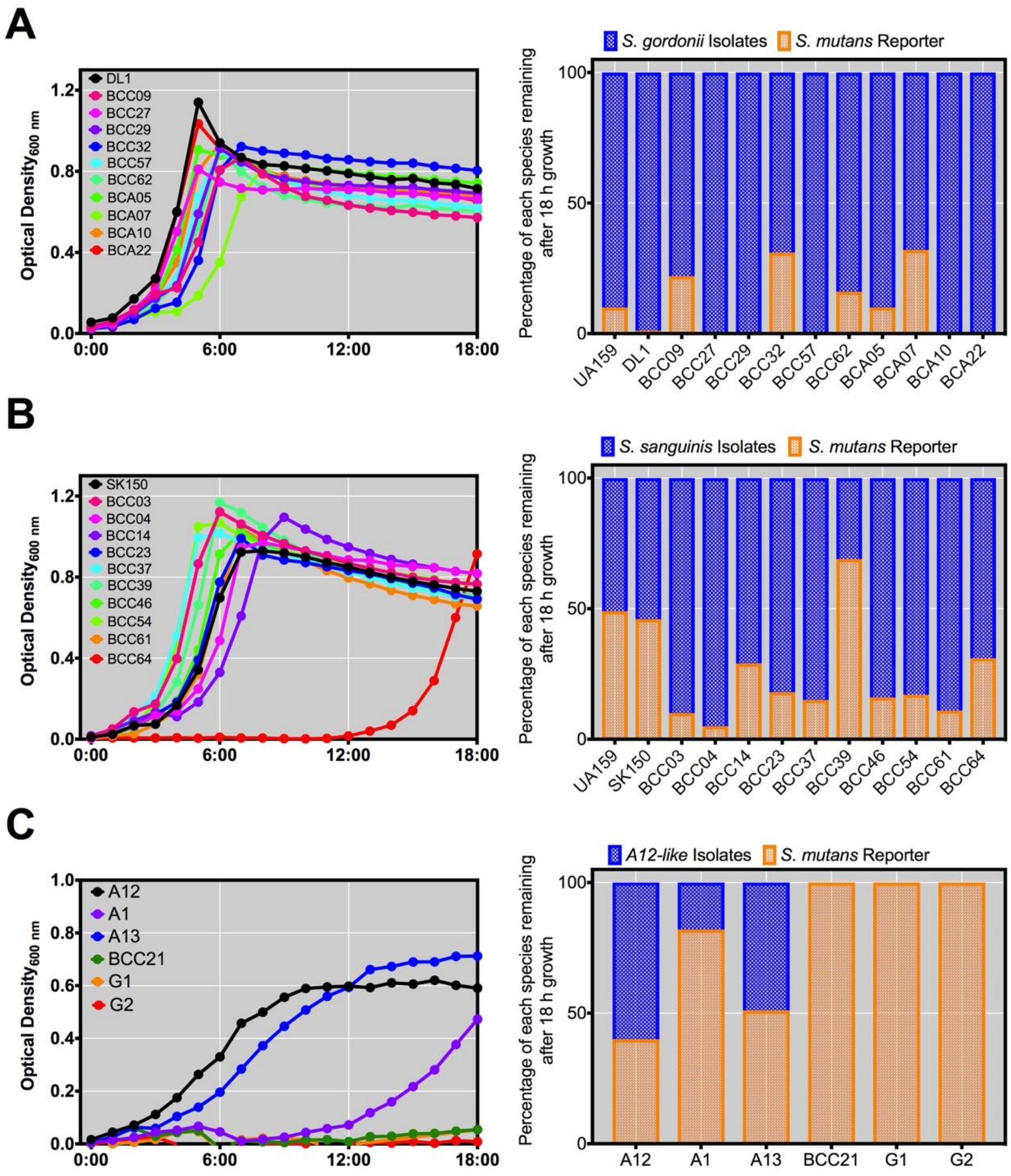

Growth curves of individual commensal streptococci isolates in CDM and CFUs after 18 h of monitoring. Growth of each individual oral isolates of either (A) *S. gordonii*, (B) *S. sanguinis* or (C) the A12-likes in CDM medium was monitored for 18 h in a Bioscreen C instrument. Cultures of all isolates were grown to mid-exponential log phase then back diluted 1/100 in CDM medium before being loaded in a Bioscreen C plate. For CFU determination, the 96 well plate was removed from the Synergy2 plate reader and individual replicates were serially diluted and plated on either BHI containing spectinomycin (pDL278 carries spectinomycin resistance) or BHI only (total CFU cell count). CFUs were enumerated to determine percentage of reporter strain remaining in the population after 18 h of monitoring.

Supplemental Figure 4

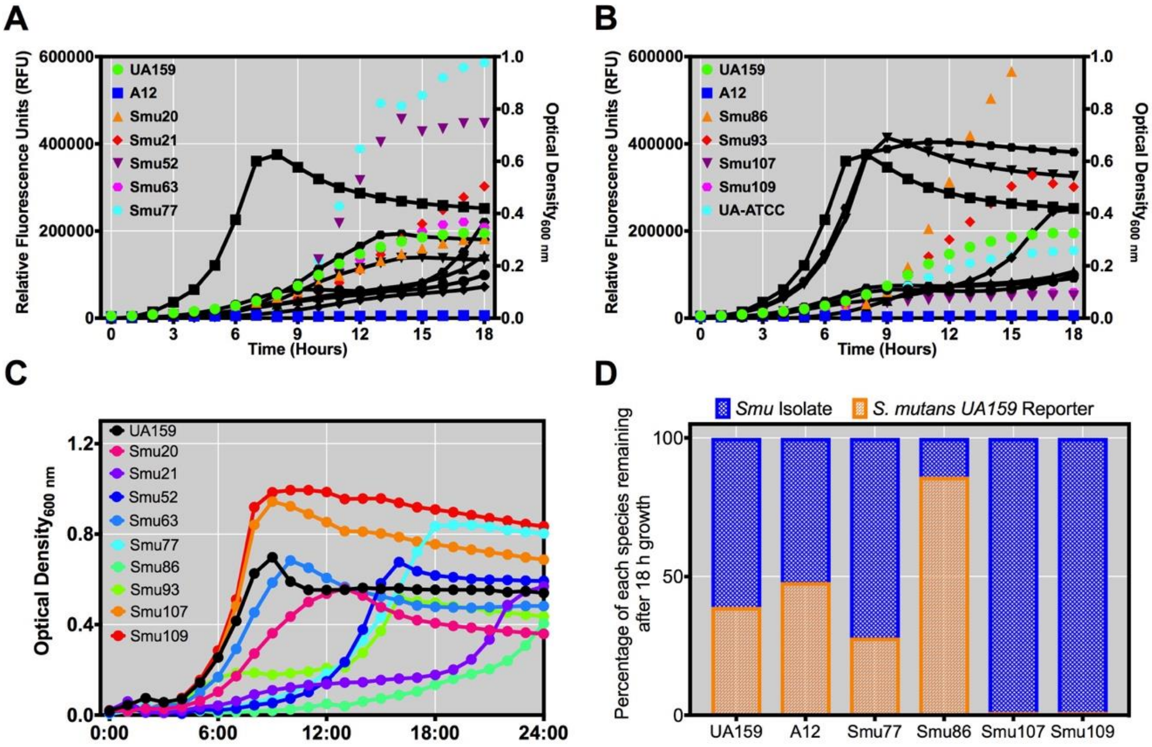

Screen of Smu Isolates cocultured with the *S. mutans* P*comX*::*gfp* Reporter. (A & B) Selected Smu isolates were screened for their ability to inhibit ComRS signaling when cocultured with *S. mutans* UA159 P*comX*::*gfp* reporter. The reporter cocultured with a reporterless UA159 served as a positive control (green dots) and coculturing with *S. sp. A12* served as a negative control (blue squares). Coculturing of *S. mutans* with other *S. mutans* isolates did not inhibit ComRS signaling activity. Cocultures with two isolates, Smu107 and Smu109, displayed less reporter activity than with the other isolates. This is most likely due to the faster growth rates and higher carrying capacity of these two isolates as seen in (C), recorded growth rates of individual isolates in a Bioscreen C instrument. Additionally, (D) CFU enumeration showed that the UA159 reporter strain consisted of < 1% of all CFUs after 18 h of monitoring, consistent with the observed decreased reporter activity.

Supplemental Figure 5

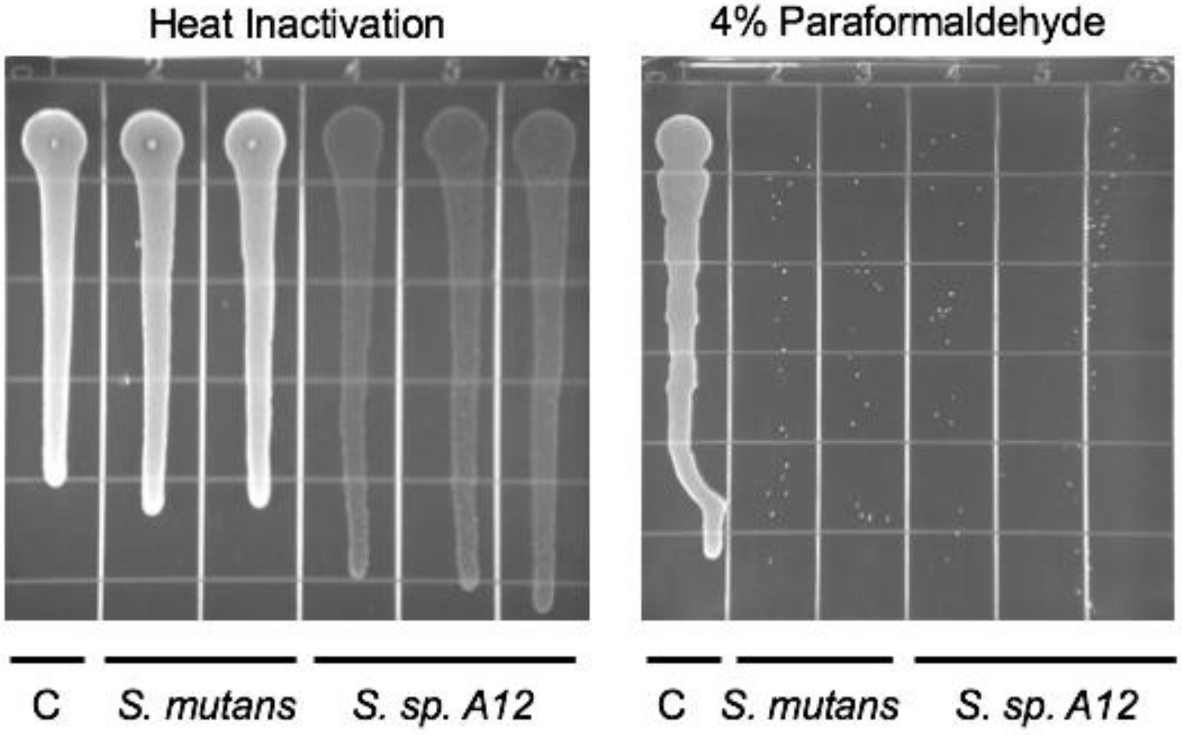

Bacterial recovery after selected inactivation treatments. 1 mL cultures of *S. mutans* UA159 or *S. sp. A12*, grown to mid-exponential log phase, were treated either by heat inactivation (0.5 h at 80˚C in benchtop heat block) or suspension in 4% paraformaldehyde PBS for 1 h. To determine effectiveness of selected bacterial inactivation treatments, 10 µL out of 1 mL treated culture were spotted on square BHI agar plates using the drop-spot method, were the cultures are spotted and then the agar plate tilted up to let the spot run down the agar plate for resolution of individual bacterial colonies for colony forming unit enumeration. A lawn of bacteria was recovered for the heat inactivation treatment while individual colonies could be seen in the 4% paraformaldehyde treatment. C = control, non-treated *S. mutans* UA159 culture, followed by treated cultures of either *S. mutans* (used as controls in the fluorescent reporter experiment shown in Figure 5) or *S. sp. A12*.

Supplemental Figure 6

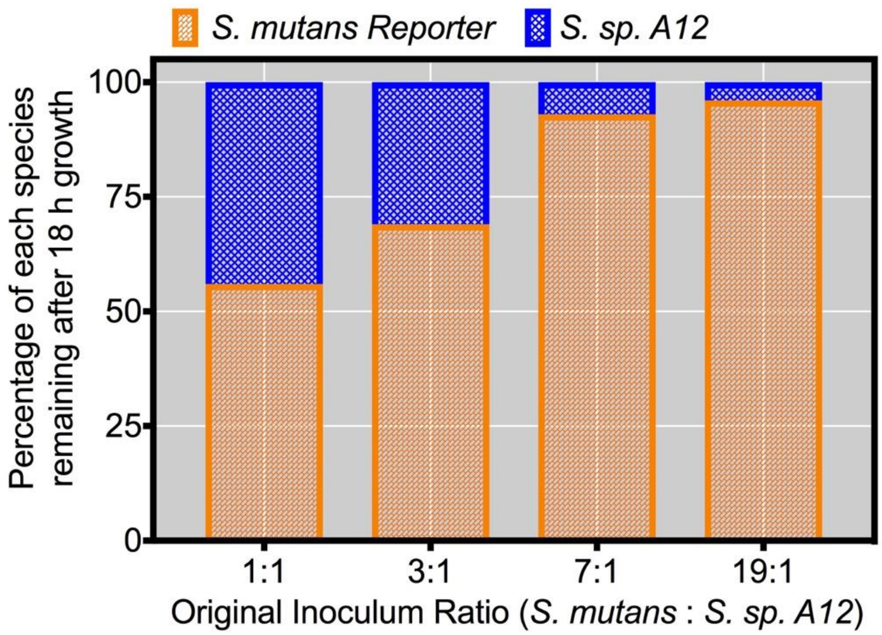

Recovery of different inoculation ratios of *S. mutans* P*comX*::*gfp* reporter to *S. sp. A12* after 18 h of growth. The *S. mutans* reporter strain P*comX*::*gfp* and commensal competitor *S. sp. A12* were inoculated in CDM growth medium at different inoculation ratios and then fluorescence of the coculture monitored in a Synergy2 plate reader for 18 h. For CFU determination, the 96 well plate was removed from the plate reader and individual replicates were serially diluted and plated on either BHI containing spectinomycin (reporter count, pDL278 carries spectinomycin resistance) or BHI only (total CFU cell count). CFUs were enumerated to determine percentage of reporter strain remaining in the population after 18 h of monitoring. Results showed that for cocultures of *S. mutans* and S. sp. A12, the population recovered after 18 h of growth remained close to the original inoculation ratio and was used in a competitor concentration experiment (Figure 5C).

Supplemental Figure 7

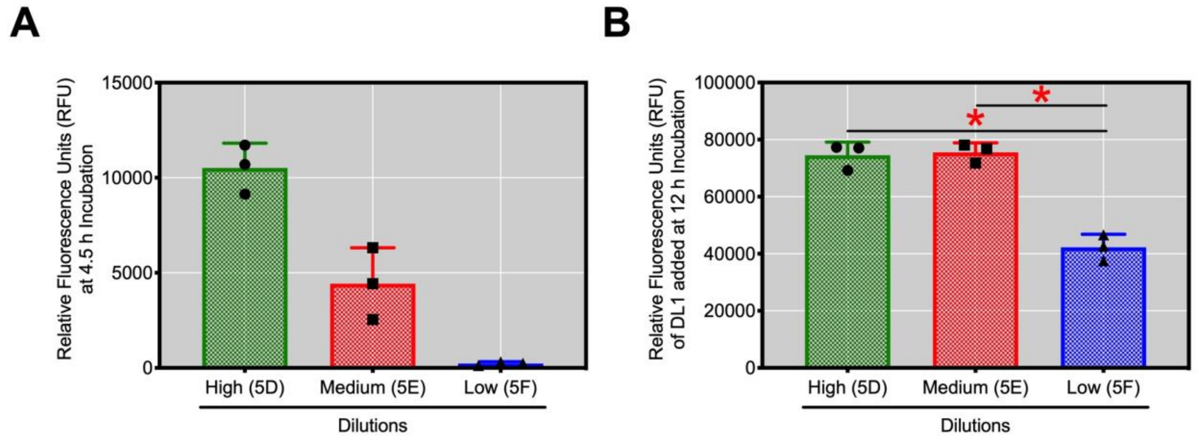

Relative Fluorescent Unit Measurements During Competitor Addition Experiments From Figure 5D-F: *S. mutans* reporter strain P*comX*::*gfp* was added at different dilutions to begin the experiment, either at a high (1:50, represented in Figure 5D), medium (1:66, represented in Figure 5E) or low (1:100, represented in Figure 5F) dilution, and activation of ComRS signaling by the reporter was monitored using a Synergy 2 multimode plate reader. (A) At 4.5 h, different levels of activation were seen, with the high dilution being activated, the medium dilution beginning to be activated and the low dilution not activated yet. At 4.5 h, either *S. mutans* UA159 (control) or *S. gordonii* DL1 (competitor) was added to the reporter strain and readings in the Synergy 2 plate reader were resumed. (B) Differences in reporter activities between dilutions when *S. gordonii* DL1 was added at 12 h into the experiment. The lower dilution, not activated for ComRS signaling at 4.5 h, showed significantly less RFUs compared to both the high and medium dilutions (Student's T-Test, * = P-value of 0.03 for both comparisons).
The experiment was performed with four biological replicates recorded in technical quadruplets.

Supplemental Figure 8

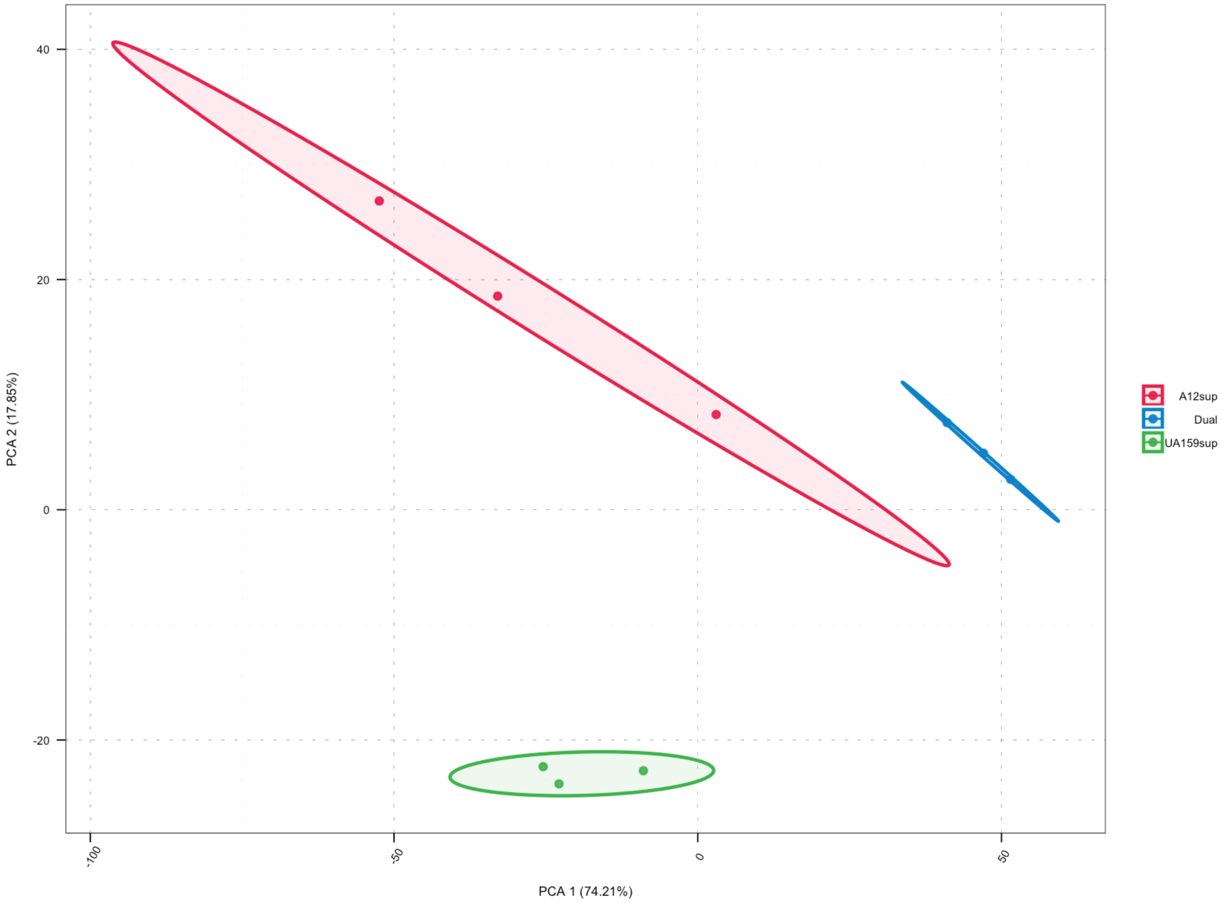

Score Plot of Principal Component Analysis on RNA-Seq Data. Principal component analysis of transcriptome profiles between three different culturing conditions of *S. mutans* UA159: in its own spent supernatant fluid (UA159sup, green), in *S. sp. A12* spent supernatant fluid (A12sup, red), and directly cocultured with *S. sp. A12* (Dual, blue).

Supplemental Figure 9

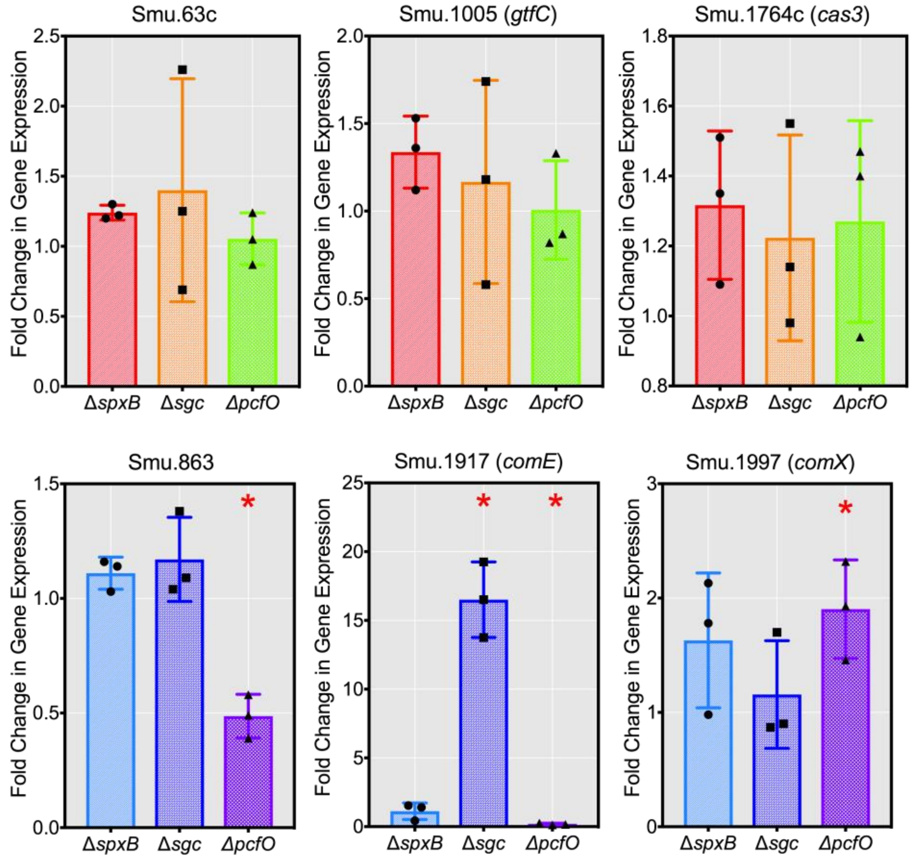

qRT-PCR of *S. mutans* differentially expressed genes during coculture with select *S. sp. A12* mutants. qRT-PCR of selected upregulated (top row) or downregulated (bottom row) genes from coculture with selected *S. sp. A12* strains. Cocultures of *S. mutans* UA159 and selected mutants were grown in CDM medium to $OD_{600\ nm}$ = 0.5 before harvest and RNA extraction. Data represents fold change in gene expression compared to *S. mutans* UA159 cocultured with the wild-type *S. sp. A12* strain. Data was normalized to internal SMU.996 and SMU.1616c controls, similar to Figure 7. Three independent cocultures were analyzed and plotted. Red star denotes statistical significance by Student's T-Test within the GraphPad Prism software, P $\leq$ 0.05.

Supplemental Figure 10

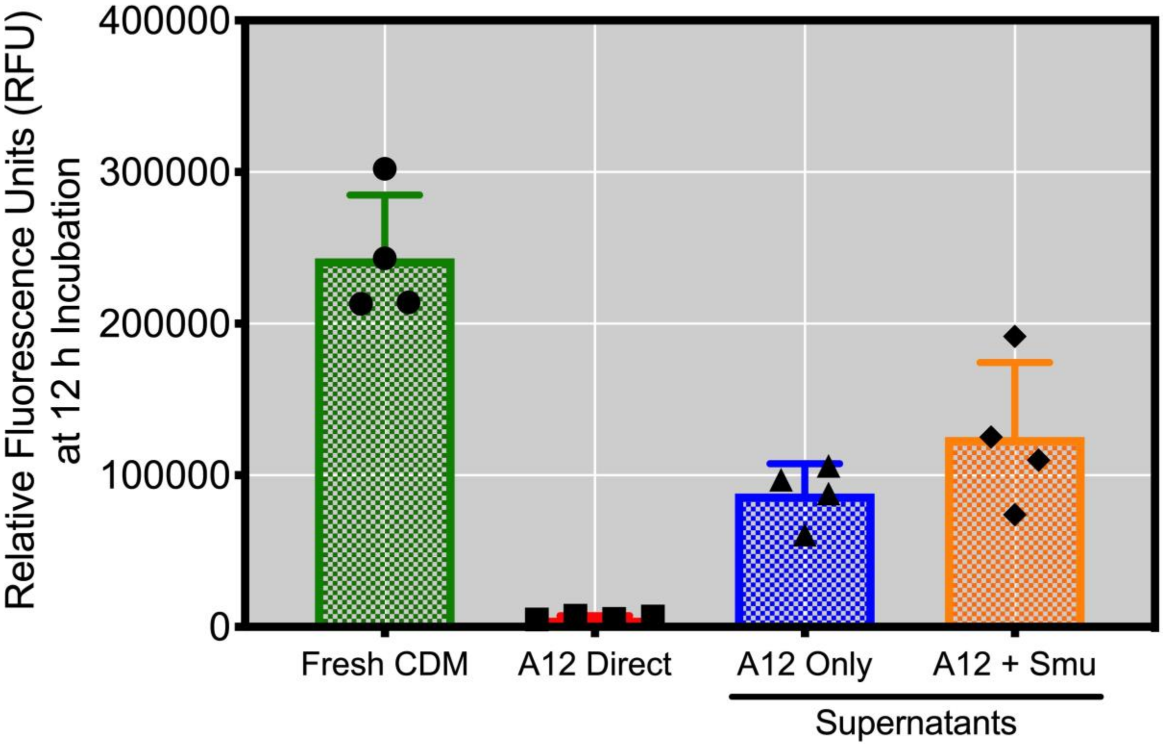

ComRS Signaling Inhibition in Commensal Supernatants. Comparison of relative fluorescent units (RFUs) after 12 h of incubation for the *S. mutans* reporter strain P*comX*::*gfp* grown within different supernatants. Fresh CDM (green bars) is inoculation of reporter strain into fresh (non-supernatant) CDM to begin the experiment as serves as a positive control. A12 Direct (red bars) is inoculation of *S. sp. A12* with the reporter strain into fresh CDM and serves as the negative control. In this experiment, two different supernatants were tested. Supernatant from *S. sp. A12* monoculture grown overnight (blue bars, similar to Figure 3A) or supernatant from a *S. sp. A12* and *S. mutans* UA159 coculture grown overnight (orange bars). Overnight cultures of selected strains where centrifuged, spent supernates removed, filter sterilized, the pH was adjusted to 7.0 and 20 mM additional glucose was added. The P*comX*::*gfp* reporter strain was then inoculated and monitored for 18 h in a Synergy 2 multimode plate reader. The experiment was performed with four biological replicates recorded in technical quadruplets.

Movie S1. S. mutans PcomX::gfp Active Cell Within a Cocultured Biofilm. Movie of the 3D rendering of a 40 µm x 40 µm area within a SYTO 42-stained (total cells, blue) *S. mutans* (P*comX*::*gfp*, green) and *S. gordonii* (P23::*dsRed*2, red) cocultured biofilm. Two P*comX*-active cells are located at/near the substratum of the biofilm, within a *S. mutans* microcolony. The image in the movie is the same as that displayed in the top panel of Figure 2B.

SUPPLEMETNAL MATERIAL REFERENCES

1. **Lau PC.**, **Sung CK**, **Lee JH**, **Morrison DA**, **Cvitkovitch DG**. 2002. PCR ligation mutagenesis in transformable streptococci: application and efficiency. J Microbiol Methods **49**:193–205.
2. **Lee K**, **Walker AR**, **Chakraborty B**, **Kaspar JR**, **Nascimento MM**, **Burne RA**. 2019. Novel Probiotic Mechanisms of the Oral Bacterium *Streptococcus sp. A12* as Explored with Functional Genomics. Appl Environ Microbiol **85**:e01335-19.
3. **Huang X**, **Palmer S**, **Ahn S-J**, **Richards VP**, **Williams ML**, **Nascimento MM**, **Burne RA**. 2016. Characterization of a highly arginolytic *Streptococcus* species that potently antagonizes *Streptococcus mutans*. Appl Environ Microbiol **82**:2187-2201.
4. **Kaspar J**, **Shields RC**, **Burne RA**. 2018. Competence inhibition by the XrpA peptide encoded within the *comX* gene of *Streptococcus mutans*. Mol Microbiol **109**:345–364.
5. **Son M**, **Ahn S-J**, **Guo Q**, **Burne RA**, **Hagen SJ**. 2012. Microfluidic study of competence regulation in *Streptococcus mutans*: environmental inputs modulate bimodal and unimodal expression of *comX*. Mol Microbiol **86**:258–72.
6. **Kaspar J**, **Underhill SAM**, **Shields RC**, **Reyes A**, **Rosenzweig S**, **Hagen SJ**, **Burne RA**. 2017. Intercellular communication via the *comX*-Inducing Peptide (XIP) of *Streptococcus mutans*. J Bacteriol JB.00404-17.
7. **Cornejo OE**, **Lefébure T**, **Bitar PDP**, **Lang P**, **Richards VP**, **Eilertson K**, **Do T**, **Beighton D**, **Zeng L**, **Ahn S-J**, **Burne RA**, **Siepel A**, **Bustamante CD**, **Stanhope MJ**. 2013. Evolutionary and population genomics of the cavity causing bacteria *Streptococcus mutans*. Mol Biol Evol **30**:881–93.
8. **Palmer SR**, **Miller JH**, **Abranches J**, **Zeng L**, **Lefebure T**, **Richards VP**, **Lemos JA**, **Stanhope MJ**, **Burne RA**. 2013. Phenotypic heterogeneity of genomically-diverse isolates of *Streptococcus mutans*. PLoS One **8**:e61358.
9. **Huang X**, **Browngardt CM**, **Jiang M**, **Ahn S-J**, **Burne RA**, **Nascimento MM**. 2018. Diversity in Antagonistic Interactions between Commensal Oral Streptococci and *Streptococcus mutans*. Caries Res **52**:88–101.
10. **Velsko IM**, **Chakraborty B**, **Nascimento MM**, **Burne RA**, **Richards VP**. 2018. Species Designations Belie Phenotypic and Genotypic Heterogeneity in Oral Streptococci. mSystems **3**:e00158-18.
11. **LeBlanc DJ**, **Lee LN**, **Abu-Al-Jaibat A**. 1992. Molecular, genetic, and functional analysis of the basic replicon of pVA380-1, a plasmid of oral streptococcal origin. Plasmid **28**:130–45.
12. **Biswas I**, **Jha JK**, **Fromm N**. 2008. Shuttle expression plasmids for genetic studies in *Streptococcus mutans*. Microbiology **154**:2275–82.
13. **Guo Q**, **Ahn S-J**, **Kaspar J**, **Zhou X**, **Burne RA**. 2014. Growth phase and pH influence peptide signaling for competence development in *Streptococcus mutans.* J Bacteriol **196**:227–36.
